# The transportome of the endophyte *Serendipita indica* in free life and symbiosis with *Arabidopsis* and its expression in moderate salinity

**DOI:** 10.3389/fmicb.2023.1191255

**Published:** 2023-06-19

**Authors:** Rosario Haro, Mónica Lanza, Marcos Aguilella, Eugenio Sanz-García, Begoña Benito

**Affiliations:** ^1^Centro de Biotecnología y Genómica de Plantas, Universidad Politécnica de Madrid, Instituto Nacional de Investigación y Tecnología Agraria y Alimentaria, Madrid, Spain; ^2^Departamento de Biotecnología-Biología Vegetal, Escuela Técnica Superior de Ingeniería Agronómica, Alimentaria y de Biosistemas, Universidad Politécnica de Madrid, Madrid, Spain

**Keywords:** *Serendipita indica*, endophyte, transportome, ENA ATPases, Na^+^ transporters, symbiosis, salinity, *Arabidopsis*

## Abstract

*Serendipita indica* is an endophytic root symbiont fungus that enhances the growth of various plants under different stress conditions, including salinity. Here, the functional characterization of two fungal Na^+^/H^+^ antiporters, SiNHA1 and SiNHX1 has been carried out to study their putative role in saline tolerance. Although their gene expression does not respond specifically to saline conditions, they could contribute, together with the previously characterized Na^+^ efflux systems SiENA1 and SiENA5, to relieve Na^+^ from the *S. indica* cytosol under this stressed condition. In parallel, an *in-silico* study has been carried out to define its complete transportome. To further investigate the repertoire of transporters expressed in free-living cells of *S. indica* and during plant infection under saline conditions, a comprehensive RNA-seq approach was taken. Interestingly, *SiENA5* was the only gene significantly induced under free-living conditions in response to moderate salinity at all the tested time points, revealing that it is one of the main salt-responsive genes of *S. indica*. In addition, the symbiosis with *Arabidopsis thaliana* also induced *SiENA5* gene expression, but significant changes were only detected after long periods of infection, indicating that the association with the plant somehow buffers and protects the fungus against the external stress. Moreover, the significant and strongest induction of the homologous gene *SiENA1* occurred during symbiosis, regardless the exposure to salinity. The obtained results suggest a novel and relevant role of these two proteins during the establishment and maintenance of fungus-plant interaction.

## Introduction

Plasma membrane-localized transporters are one of the primary links between the external environment and the metabolic pathways inside all living cells. The lipophilic nature of membranes makes the existence of these type of proteins essential, which, arranged along the lipid bilayer, allow ions and hydrophilic substances to cross the cell barrier. Their functionality not only ensures the acquisition of the nutrients necessary for cellular life, but also allows to get rid of products that can be toxic to the cell, whether derived from cellular metabolism or substances from outside that have somehow entered the cell. In fungi, the most complete study carried out to date on the identification and functional characterization of the set of transporters has been done in the yeast *Saccharomyces cerevisiae* (Paulsen et al., 1998; De Hertogh et al., 2002), whose transportome accounts for approximately 5% of the cellular proteome (Brohée et al., 2010). More recent studies have also partially addressed the analysis of transporter families existing in mycorrhiza, fungi that establish stable mutualistic symbiotic relationships with plants (Casieri et al., 2013; Courty et al., 2016; Garcia et al., 2016). In these fungi, there is increasing interest in functional knowledge of the transporter proteins, since some of them are responsible for the acquisition of nutrients from the soil that can be exchanged with the host plant, so their activity may have a practical relevance for agriculture and the environment. Advances in the development of sequencing programs of complete genomes help to acquire wealthy information on the pool of transporters that each organism possesses. Interestingly, phylogenetic analysis of the set of transporters of different organisms shows that throughout evolution, various families of transporters have shared structural features and modes of action that have been conserved among different organisms; and despite in most cases the substrate they transport has not yet been determined, their conservation supports a functional importance of this group of proteins.

Although fungi are equipped with a variety of transporters, not all of them are needed in all environmental condition that they can naturally cope with, so a fine regulation of their gene expression must take place. This may be the case for some endophytic fungi that alternate between two completely different lifestyles and environments: soil, a very changeable and sometimes stressful environment in terms of nutrient availability and pH conditions; versus the not-so-changing environment of plant tissues, in which they grow during plant colonization.

*Serendipita indica* is an example of a root-colonizing endophytic fungus that promotes the growth of various plants and helps them withstand different types of biotic and abiotic stresses (Unnikumar et al., 2013). The beneficial effects of this fungus are very diverse, although the mechanisms underlying these effects are still pending to be totally unraveled. The positive response of the plant may be the result of an improvement in nutrition mediated by the fungus, as has been demonstrated for phosphorus, magnesium, iron and sulfur (Bajaj et al., 2018; Prasad et al., 2019; Narayan et al., 2021; Verma et al., 2022). However, additionally, it can also imply changes in the hormonal status of the plant due to the production of phytohormones by the fungus itself or by the plant in the presence of the fungal symbiont resulting, in any case, in better plant growth. In fact, *S. indica*, as other endophytic fungi, is shown to produce phytohormones itself that may contribute to the effect on plant growth (Xu et al., 2018; Meents et al., 2019).

One of the most important abiotic stresses that affect plants is salinity. It is well known that salt stress comprises ion imbalance, which can cause nutritional stress, mainly for K^+^; hyperosmotic stress, which affects the uptake and movement of water; and oxidative damage due to the formation of superoxide radicals. A wealth of literature supports that these three aspects are essential for triggering the response of the plant to salinity; however, whether any of them has a prevailing importance is still unsolved (Isayenkov and Maathuis, 2019).

Undoubtedly, the contribution of transporters involved in the uptake and distribution of Na^+^ but also K^+^ within the plant would play a crucial role in salt stress responses.

In a previous work, we studied the beneficial interaction of *S. indica* with *Arabidopsis* plants grown exposed to saline conditions, demonstrating that fungal colonization reduced the Na^+^ contents of the plant (Lanza et al., 2019), suggesting the performance of the endophyte as a Na^+^ barrier for the plant. However, Na^+^ content reduction could alternatively be caused by the activity of some fungal or plant effector proteins, among which the Na^+^ transporters could be good candidates. On the fungal side, the main transport systems known to be essential for salt tolerance are ENA ATPases (Rodríguez-Navarro and Benito, 2010), *S. indica* has two, SiENA1 and SiENA5, whose function has been recently characterized (Lanza et al., 2019). These are P-type ATPases located in the plasma membrane that pump Na^+^ (and some of them also K^+^) out of the cells, alleviating the toxic and osmotic cellular stress. Hypothetically, the activity of Na^+^ pumps of endophytic fungi surrounding the roots could directly extrude and alleviate Na^+^ from plants. Other Na^+^ transporters that have been shown to play a relevant role in salinity tolerance in fungi by excluding Na^+^ from the cytosol are Na^+^-H^+^ antiporters. The so-called NHA, are located in the plasma membrane and extrude Na^+^ out of the cell; the so-called NHX show abundance in the early endosome and pre-vacuolar vesicles shuttling Na^+^ into these organelles (Ariño et al., 2018). Both systems are pH-dependent because they use the H^+^ gradient generated by H^+^ ATPases to move the Na^+^ (and/or K^+^) across membranes (Ariño et al., 2018).

Since 2011 the genome of *S. indica* has been completely sequenced (Zuccaro et al., 2011), which undoubtedly has allowed faster progress in the study of the genes and functions of the proteins encoded by them. At that time and by comparing the automatically translated proteome of the fungus with the Transporter Classification Database (TCDB),^1^ the automatic annotation of *S. indica* transporters was performed.^2^ For the first time, a preliminary reference was reported on fungus transporters that were regulated during root colonization (Zuccaro et al., 2011). There, they manually identified 64 of the 262 annotated transporters that were regulated during the saprotrophic or biotrophic stages of colonization.

Here we present a complete “Transportome” of *S. indica* defined on the basis of homology with evolutionary and structurally related proteins and on functional information obtained from orthologous proteins. Transcriptional changes of genes that constitute the transportome have been studied under two different conditions, free-living and in symbiosis with *A. thaliana*, exposed or not to moderate salt stress produced by 50 mM NaCl. In addition, the functional characterization of the two Na^+^-H^+^ antiporters, SiNHA1 and SiNHX1, has also been addressed to find out if, as in other fungi, they play a role in the salinity tolerance of *S. indica*. The results presented here showed that moderate salinity hardly affects the expression of the global transportome of *S. indica*. However, the transition from the free lifestyle to symbiosis involves a reprogramming of numerous fungal transporters necessary to adapt to a completely different environment. Interestingly, the gene expression results shown here suggest that ENA ATPases, *SiENA5* and *SiENA1*, are two relevant transporters to deal with salinity under free living conditions and to establish and maintain symbiosis, respectively.

## Materials and methods

### Plant, fungi, bacteria, and yeast strains

*Col-0 Arabidopsis* plants and *S. indica* strain DSM 11827, DSMZ, Braunschweig, Germany, were used throughout this study. The *Escherichia coli* strain DH5α was routinely used for the propagation of plasmids. The *S. cerevisiae* strain used was AXT3K [*Mat* **a** *ade2 ura3 trp1 ena1-4::HIS3 nha1*:*:LEU2 nhx1::KanMX4*], in which the Na^+^ efflux systems ENA1-4, NHA1 and NHX1 are absent (Quintero et al., 2002). This yeast mutant was used for complementation studies using arginine phosphate (AP) medium (Rodriguez-Navarro and Ramos, 1984) or YPD medium in the presence of variable NaCl and KCl concentrations. *Arabidopsis* seeds were surface sterilized for 5 min in 5% sodium hypochlorite and finally washed with sterile water for 1 h. After stratification for 3 days at 4°C in the dark, the seeds were germinated in PNM-agar plates placed vertically for 1 week. The plants were grown under control conditions in a greenhouse with an 8-h light/16-h dark period at 22°C. *S. indica* was grown in the minimal PNM medium (Lahrmann et al., 2013) supplemented with 2% glucose. The saline stress experiments of the fungus in free living conditions were performed on AP medium supplemented with the 5 mM KCl and in the presence or absence of 50 mM NaCl.

### Salt stress experiments in the infected plants

The experiments carried out in *Arabidopsis*, including fungal inoculation and transfer to saline conditions, were carried out simultaneously. For that, 2-weeks-old seedlings were transferred to sterile square Petri dishes containing PNM medium without glucose supplementation in the presence or absence of 50 mM NaCl. Thereafter, they were directly inoculated with 100 μL of a chlamydospore suspension (5 × 10^5^ mL^−1^ in 0.002% Tween20). Incubation was performed by placing the plates vertically in a Conviron phyto-chamber with a day/night cycle of 16/8 h (light 21 intensity: 110 μmol m^−2^ s^−1^) at a temperature of 22/18°C. After 1 week, the plants were individually dissected into roots and shoots for further analysis, e.g., total RNA extraction or cation quantification.

### Analysis of root colonization by *Serendipita indica*

To check for root colonization, 5 root samples were selected randomly from the plants. The samples were softened in 10% KOH solution for 15 min, acidified with 1 N HCl for 10 min and finally stained with 0.02% Trypan blue overnight (Jogawat et al., 2016). The samples were de-stained with 50% Lacto-phenol for 1–2 h, followed by 3–4 washing steps with ethanol 100% and suspension in 60% glycerol prior to their light microscopic inspection.

### Cloning of *SiNHA1* and *SiNHX1* cDNAs and yeast complementation

The full-length *SiNHA1* and *SiNHX1* cDNAs were obtained by RT-PCR from total RNA extracted from *S. indica* using specific primers that amplified DNA fragments containing the respective predicted START and STOP codons (for *SiNHA1*, SiNHA1-ATG primer 5´-TTCCTGGATGGGCTTTTATC-3′, and SiNHA1-STOP primer 5′-GGGCATCGATTGATGAAGAC-3′ were used and, for SiNHX1, SiNHX1-ATG primer 5′-GCCAATATGCTCGAAAAGAGA-3′, and SiNHX1-STOP primer 5′-TAGAACGGAACAAGGAAACTG-3′ were used). After cDNA integrity verification by sequencing, the obtained fragments were cloned into the *Xba*I and *Kpn*I sites and *EcoR*I sites, respectively, of the yeast expression vector pYPGE15 (Brunelli and Pall, 1993). Yeast transformation was carried out as described in Elble (1992). Yeast transformants expressing SiNHA1 and SiNHX1 transporters were analyzed for NaCl tolerance by Drop tests that were carried out by inoculating cell suspensions of three serial 10-fold dilutions in different solid medium plates containing variable NaCl concentrations.

### Identification of non-annotated transporters of *Serendipita indica* by phylogenetic comparison with two fungal transportomes

Based on the evolutionary conservation of structural and functional features of proteins, we first compared the annotated transportome of *S. indica* with the well-annotated and extensively studied transportome of the ascomycete *S. cerevisiae* (Sc); and with that of a more phylogenetically related basidiomycete *Cryptococcus neoformans* (Cn), both available in TransportDB 2.0. MMseqs2 software (Mirdita et al., 2019) was used to perform the clustering of the *S. indica, S. cerevisiae*, and *C. neoformans* transporters according to the similarity of the protein sequences. The “bidirectional coverage” mode was used with a coverage of 80%, as it is recommended for clustering large multidomain proteins. From the alignment results, several clusters of proteins were obtained that included: (i) representatives of the three fungi; (ii) representatives of Sc and *S. indica*; (iii) representatives of Cn and *S. indica*; (iv) representatives of Sc and Cn; (v) representatives only of Sc; and (vi) representatives only of Cn. Those transporters of *S. indica* included in any of the first three clusters were directly considered to be part of the transportome. Representative transporters from *S. cerevisiae* or *C. neoformans* included in any of the last three clusters in which no representative proteins of *S. indica* were identified, were used for a BLASTp search (Altschul et al., 1990) against the complete proteome of the fungus with the idea of retrieving non-annotated transporters in JGI. The BLASTp results were filtered by hits that had an identity greater than 25% and a coverage greater than 50%. DeepTMHMM software (Hallgren et al., 2022) was used to predict the number of transmembrane segments. A functional analysis of the new candidates was also performed by classifying them into families and predicting domains with Pfam (Mistry et al., 2021).

### Identification of non-annotated transporters of *Serendipita indica* by EggNOG analysis of the *Serendipita indica* proteome

EggNOG (Evolutionary Genealogy of Genes: Non-supervised Orthologous Groups)^3^ is a database of relationships between orthologs, which provides functional information and evolutionary relationships of genes from COG (Cluster of Orthologous Groups) comparisons (Huerta-Cepas et al., 2019). Analysis of the annotated full proteome of *S. indica* by this database (which comprised a total of 11,198 fungal proteins), retrieved only 7,984 hits. The screening of the keywords related to transporters and channels: “transport/er,” “efflux,” “uptake,” “exporter,” “importer,” “pump,” “permease,” “exchange/r,” “channel,” “carrier,” “influx,” “symporter,” “antiporter” and “translocator”; identified 218 hits that were not previously annotated in the JGI transportome. The analysis of COG description proposed 91 protein candidates to be considered as transporters. Following these two different strategies and after revising the protein length, the presence and number of transmembrane fragments (TM) and EuKaryotic Orthologous Groups (KOG) descriptions of the hits obtained, a total of 80 new transporters were identified.

### Total RNA extraction for RNA-seq

For fungal expression analyses in free living conditions, *S. indica* was previously grown in liquid AP medium supplemented with 5 mM KCl and then transferred to fresh AP medium with or without 50 mM NaCl. Fungal samples were taken and immediately frozen in liquid nitrogen after the incubation at 25°C for 1.5 h, 7, and 14 days (Supplementary Figure S1). Fungal-inoculated plant root samples were obtained at 1.5 h, 7, and 14 days after inoculation and growth in the presence or absence of 50 mM NaCl. The first 4–5 cm of roots starting from the seed were excised, and immediately frozen in liquid nitrogen. Either fungal or fungus-inoculated plant root samples were crushed to a fine powder in liquid nitrogen with mortars and pestles. Total RNA was extracted with TRIzol reagent (Invitrogen) and on-column DNase digestion was performed according to manufacturer’s protocol. RNA was purified by passage through Nucleospin RNA Plant columns (Mackerey-Nagel). Triplicates of control and the different saline treatments were used for library construction and RNA-seq analysis. The quality of the RNA samples was checked employing an Agilent 2,100 Bioanalyzer. Those RNA samples with a RNA Integrity number (RIN) >7.5 and rRNA 28S/18S ratio > 1.5 were selected for further analysis.

### Library preparation and analysis pipeline

*Serendipita indica* and *Arabidopsis* cDNA libraries from control and saline treatments were prepared by the BGI Genomics Service, who also performed the RNA-seq experiment and basic bioinformatics processing of the obtained data, including quality control, adaptor sequence delition, reads alignment to the corresponding reference genomes, obtaining metrics for gene and transcript expression, and approaches for detecting differential gene expression. Applying their RNA-seq pipeline, filtered sequences reads were mapped to the reference *S. indica* genome^4^ using HISAT (Kim et al., 2015). To calculate normalized gene expression values using the FPKM (Fragments Per Kilobase per Million mapped reads) metrics that allow estimation of gene abundance, StringTie (Pertea et al., 2015) was used to reconstruct transcripts, and Cuffcompare (a Cufflinks tool; Trapnell et al., 2012) to compare reconstructed transcripts to reference annotation, and differential gene expression analysis was done using the DEseq2 algorithm with a cut-off value of an adjusted *p* < 0.05 (Love et al., 2014). Along with the adjusted *p* value [false discovery rate (FDR)] of <0.05 an absolute differential expression of log2 fold change (FC) ≥1 was chosen to select differentially expressed genes (DEGs).

### Transcriptomic analysis of *Serendipita indica*

The transcriptomic analysis was carried out using an Illumina RNA-seq transcriptome profiling from total RNA of *S. indica* cultures grown in AP minimal medium in the absence or presence of 50 mM NaCl (free-living conditions) and from total RNA of *Arabidopsis* roots previously infected with spores of *S. indica* (under symbiosis). Regarding the transcriptional analysis of fungus growing in free living conditions, 93% of on average of 50,000,000 of total clean reads mapped the fungal reference genome.^5^ A total of 11,198 genes were identified in the *S. indica* genome, being 11,106 out of the known genes and only 92 of them are novel genes (Supplementary Table S1). As expected, very different values of the total mapping ratio to the fungal reference genome were detected for the fungus growing on plant roots (Supplementary Table S1). Thus, out of an average of 50,000,000 total clean reads sequenced from the root infected samples, only 2–3% mapped the reference genome of the fungus, matching most of the clean reads to the plant genome.

Principal Component Analysis interestingly showed two independent clusters: one cluster was formed by the samples of free-living conditions, regardless to the exposure to salinity and the samples of the early stage of symbiosis (after 1.5 h of inoculation); the other cluster was formed by the samples of established symbiosis (after 7 and 14 dpi). These results indicated that the largest source of variation was the interaction of the fungus to plant roots with respect to free living conditions and not the saline treatment revealing that the establishment and fungal growth in symbiosis caused the most important transcriptional response of *S. indica* observed in this study (Supplementary Figure S2). To reflect the gene expression correlation between samples, the Pearson correlation coefficients were calculated for all gene expression levels between each two samples and these coefficients were reflected in the form of heat maps, shown in Supplementary Figure S3. All the samples were also hierarchical clustered by the expression level of all genes (Supplementary Figure S3). The results showed that only five RNA samples out of the 36 analyzed (FL1.5 h1; FL1.5 hNaCl1; FL7d2; FL7dNaCl3; and S1.5h2), were biased within the triplicates. This point was considered for subsequent differential expression analysis.

## Results

### Functional characterization of SiNHX1 and SiNHA1 of *Serendipita indica* and their role in Na^+^ homeostasis

Apart from ENA ATPases, Na^+^-H^+^ antiporters are known Na^+^ transport systems that are involved in salt tolerance in fungi (Ariño et al., 2018). Homologs to fungal NHX1 and NHA1 transporters (from now on, SiNHX1 and SiNHA1) were identified in the *S. indica* genome database by a BLAST search using the amino acid sequence of *S. cerevisiae* NHX1 (GenBank accession number: KZV12696.1) and NHA1 (GenBank accession number: CAA97709.1). The corresponding genes (access number PIIN_0302 for *SiNHX1* and PIIN_01646 for *SiNHA1*) were identified in the different contigs (PIRI_contig_0056 and PIRI_contig_0022, respectively). The gene structure indicated that Si*NHX1* and Si*NHA1* consist of 6 and 14 exons that encode for two proteins of 675 and 809 amino acids, respectively. Interestingly, the cDNA amplification of *SiNHX1* from total RNA of the fungus, either grown in the presence or absence of NaCl, rendered a fragment of 2025 base pairs which differed from the proposed automatically annotated gene included in the JGI (1953 base pairs encoding for 651 amino acids, see Supplementary Figure S4). The multiple sequence aligment comparison of the protein deduced from the obtained cDNA with other fungal NHX1 proteins showed that the length of the SiNHX1 deduced from the cDNA matched perfectly with the homologous proteins, suggesting that the identified discrepancy (there was an insertion of a T at the position 1,000 of the ATG in the amplified sequence) is due to an incorrect automatic annotation.

**Figure 1:**
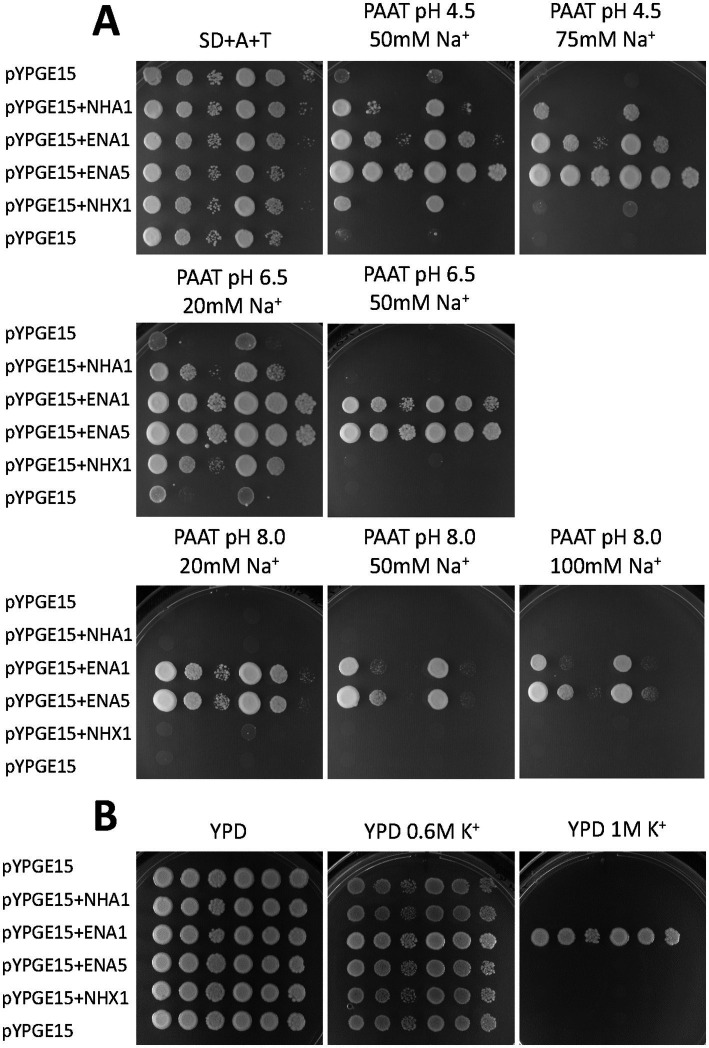
Functional complementation of Na^+^ transport-defective mutants of *Saccharomyces cerevisiae* with *Serendipita indica SiNHA1* and *SiNHX1*. A drop test assay was performed by placing 5 μl of 10-fold serial dilutions of suspensions of the mutant AXT3K transformed with SiNHA1 and SiNHX1 cDNAs cloned in the shuttle vector pYPGE15 on a solid medium. The growth of the transformants expressing the *S. indica* antiporters was compared with that of transformants expressing the fungal ENA ATPases SiENA1 and SiENA5 or the empty vector. The growth was studied in AP **(A)** or YPD **(B)** media (see Materials and methods) at different pH values and in the presence of varying concentrations of NaCl and KCl as indicated.

The functional characterization of the *S. indica* SiNHA1 and SiNHX1 was carried out by heterologous expression in yeast mutants. For that, the corresponding full-length cDNAs were cloned into a yeast expression vector and expressed in the yeast Na^+^ sensitive mutant AXT3K (see Materials and methods). The complementation assay was carried out by a drop test, comparing the ability of these two proteins to restore the growth of the yeast defective mutant in the presence of NaCl and at different pH values, in comparison to that of the previously described SiENA1 and SiENA5 Na^+^, K^+^ ATPases (Lanza et al., 2019). As shown in Figure 1, at pH 4.5, SiNHX1 was able to slightly restore the growth of the mutant in the presence of 50 mM NaCl whereas SiNHA1 enhanced the growth of the yeast mutant even in the presence of 75 mM NaCl. At pH 6.5, both transporters restored the growth of the yeast mutant only in the presence of 20 mM NaCl. Neither of them could restore yeast mutant growth at higher NaCl concentrations like the two SiENA ATPases did, nor could they restore the mutant growth at any salt concentration at pH 8.0 (Figure 1). The yeast complementation at high KCl concentrations showed no growth recovery of any of the *SiNHX1* and *SiNHA1* transformants, ruling out that any of these two transporters functioned as K^+^ efflux systems. From all these results it must be concluded that SiNHX1 and SiNHA1 function as Na^+^ transporters. Maybe, the only moderate complementation observed at high NaCl concentration could be due to heterologous expression in yeast mutants that does not always accurately reproduce the kinetic characteristics or the level of activity that the proteins have in the organism itself. Their actual contribution to salt tolerance remains to be determined by obtaining the respective mutants in *S. indica*.

### Building up the *Serendipita indica* transportome

To further investigate which are the active players of the fungus during the symbiotic interaction under saline conditions, in particular, those transporters that may be involved in Na^+^ fluxes under these conditions, a comprehensive transcriptomic analysis of the fungus was carried out by RNA-seq. However, as a previous step to this study, it was necessary to establish which are the proteins that comprise the set of transporters of *S. indica*.

According to the *S. indica* genome collected at the JGI, the inventory of automatically annotated transporters resulted in 684 genes of the 11,198 total genes that this fungus possesses.^6^ Despite generally matching the number of transporters in the proteome of other fungi, the functional characterization of some *S. indica* transporters such as two magnesium transporters, PiMgT1 and PiMgT2 (Prasad et al., 2019), or two K^+^ channels SiTOK1 and a SKC-like channel (Conchillo et al., 2021), whose genes were not annotated in the fungal genome has been recently published. This led us to review and try to build a more accurate and comprehensive transportome of *S. indica*. For that, we have followed two different approaches: a phylogenetic comparison of the full *S. indica* proteome with the fungal transportomes of *S. cerevisiae* and *C. neoformans* compiled in TransportDB 2.0^7^ (Elbourne et al., 2017); and an analysis based on orthology relationships and functional annotations using the eggNOG database (Huerta-Cepas et al., 2019; see Materials and methods section). Following these two different strategies, a total of 80 new transporters were identified.

**Figure 2:**
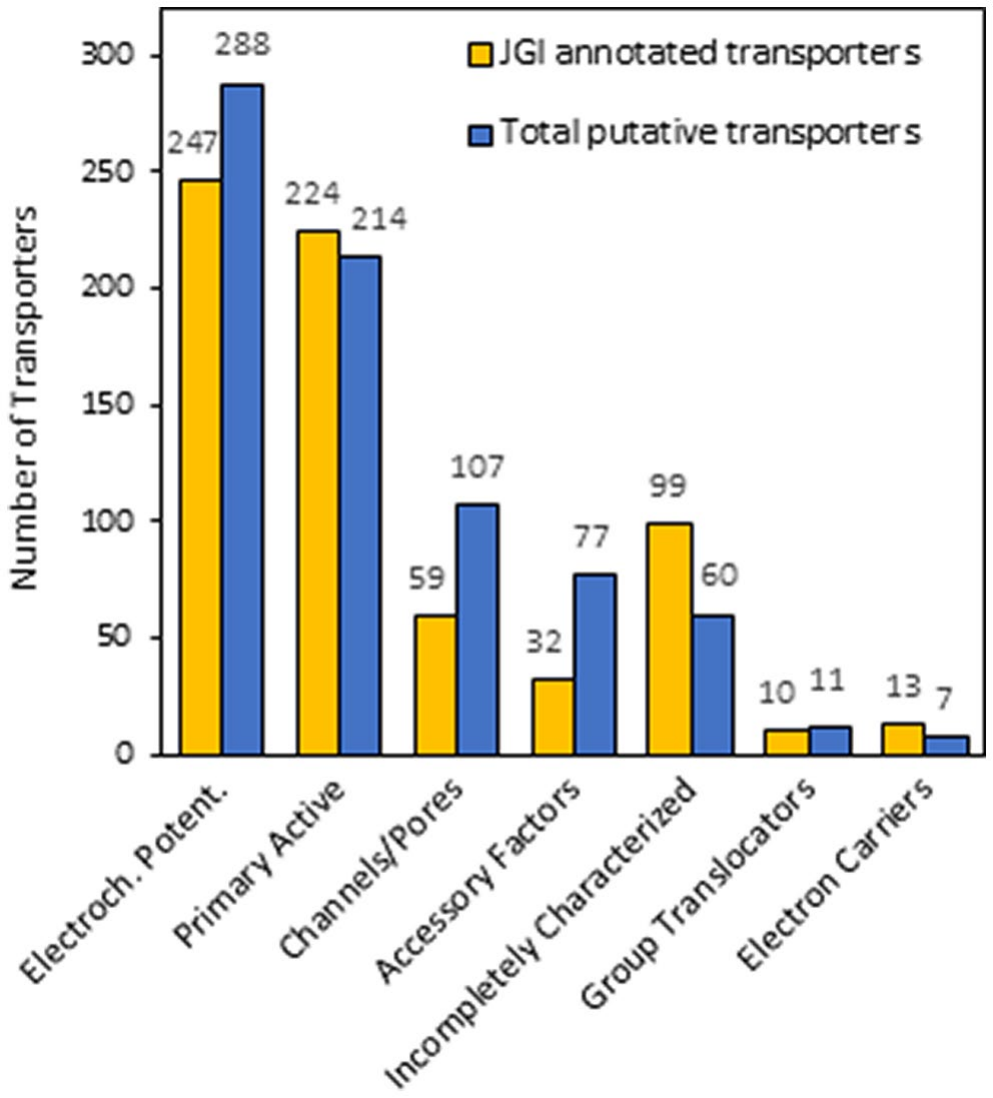
Number of *Serendipita indica* transporters grouped according to Transporter Classification Database (TCDB). The bar graph depicts the number of transporters initially annotated in the JGI (yellow bars), the number of total transporters identified in this work (blue bars). The main classes of transporters according to TCDB shown from left to right are Electrochemical potential-driven transporters, Primary active transporters, Channels/pores, Accessory factors, Incompletely characterized transport systems, Group translocators and Transmembrane electron carriers.

**Figure 3:**
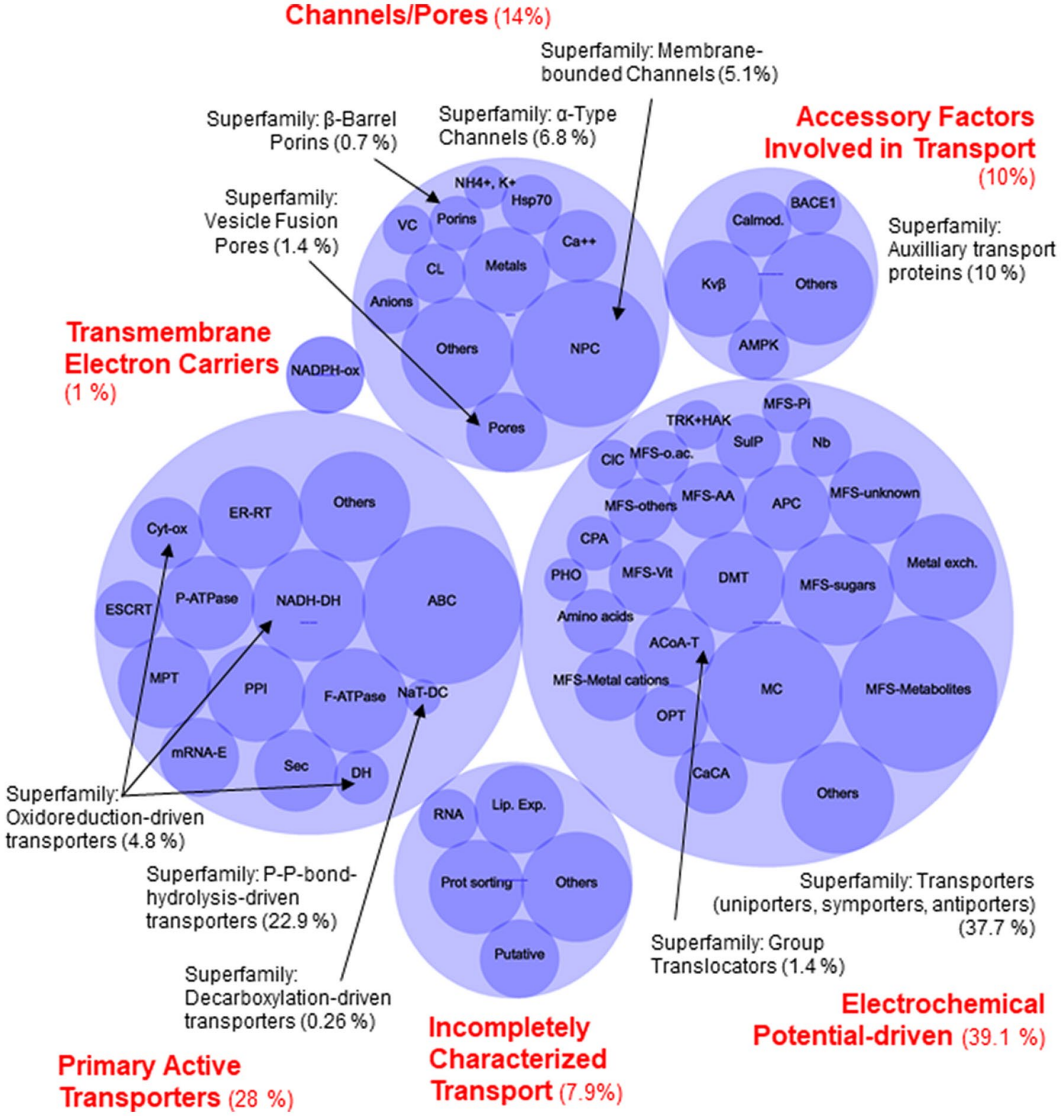
Circular packing scheme of *Serendipita indica* transportome of the 764 identified proteins. Blue light circles with red labels correspond to the main classes of Transporter Classification Database (TCDB). Blue dark circles represent to the main families of transporters. Black labels correspond to the superfamilies and the arrows point to the families which belongs to the smaller superfamilies in each class. Channel/Pores Class (107 proteins, 14%): Superfamily of Membrane-bounded Channels (39 protein, NPC (Nuclear Pore Complex Family) 5.1%); Superfamily of β-Barrel porins (5 proteins, 0.7%); Superfamily of Vesicle Fusion Pores (11 proteins, 1.4%); and Superfamily of α-Type Channels (52 proteins, 6.8%, which includes: VC, Voltage-gated Ion Channel Family; Cl^−^, Calcium-dependent Chloride Channel Family; Metals; Anion; Mg^2+^; Ca^2+^, NH_4_^+^ or K^+^ channels families; Others). Transmembrane Electron Carriers Class (1%), 7 proteins of Transmembrane 1-electron transfer carriers Superfamily, NADPH oxidase Family. Primary Active Transporters Class (214 proteins, 28%): Superfamily of Oxidoreduction-driven transporters (4.8%, 37proteins, which include: NADH-DH, the H^+^ or Na^+^-translocating NADH Dehydrogenase Family; Cyt-ox, Cytochrome Families; DH, others Dehydrogenase Families), Superfamily of Decarboxylation-driven transporters (2 proteins, 0.26%, NaT-DC, the Na^+^-transporting Carboxylic Acid Decarboxylase Family) and the Superfamily of the P–P-bond-hydrolysis-driven transporters (175 proteins, 22.9%) (MPT, Mitochondrial Protein Translocase Family; F-ATPase, F-type ATPase Family; ER-RT, Endoplasmic Reticular Retrotranslocon Family; PPI, Peroxisomal Protein Importer Family; ESCRT, the Endosomal Sorting Complexes Required for Transport Family; P-ATPase, P-type ATPase Family; Sec, General Secretory Pathway Family; ABC, ATP-binding Cassette Family; mRNA-E, the Nuclear mRNA Exporter Family). Incompletely Characterized Transport Class (60 proteins, 7.85%): 11 proteins are Putative transport proteins (Putative) and 49 proteins are recognized transporters of unknown biochemical mechanism (Lipid-translocating Exporter (LTE) Family; RNA, RNA exporter Families; Prot.Sorting, Retromer Assembly protein sorting Families). Electrochemical Potential-driven Class (299 proteins, 39.1%): Group Translocators Superfamily (11 proteins, 1.44%, ACoA-T, the Acyl-CoA Thioesterase Family) and the biggest Superfamily of Porters (uniporters, symporters, antiporters) (288 proteins, 37.7%, OPT, Oligopeptide Transporter Family, TRK-HAK, K^+^ Transporter Families; CPA, Monovalent Cation:Proton Antiporter Family; PHO, Phosphate Permease Family; SulP, Sulfate Permease Family; ClC, Chloride Carrier/Channel Family; CaCA, Ca^2+^:Cation Antiporter Family; APC, Amino Acid-Polyamine-Organocation Family; Amino acids, several families of amino acids transporters; DMT, Drug/Metabolite Transporter Superfamily; Nucleobase, nucleoside o nucleobase transporter Families; MC, Mitochondrial Carrier Family; MFS, Major Facilitator Superfamily; and Metal Exchangers Families). Accessory Factors Involved in Transport Class (77 proteins, 10%), mainly the Auxiliary transport proteins Superfamily: GPC, the G-Protein αβγ Complex Family; Kvβ, the Voltage-gated K^+^ Channel β-subunit Family; Calmod., the Calmodulin Calcium Binding Protein Family; AMPK, the 5′-AMP-activated protein kinase Family; BACE1, the β-Amyloid Cleaving Enzyme Family. The circles named “Others” group individual transporters or families with a very low number of transporters (see Supplementary Table S2 for details).

To confirm that the set of protein candidates identified above were related to transport and to ascribe them to one of the large evolutionary and structurally related families or superfamilies of transporters, a BLAST search of the new 80 proteins in the Transporter Classification Database (TCDB) was performed (accessed in December 2022).^8^ This database is a reference for the classification of membrane transporter proteins of all living organisms based on both functional and phylogenetic information. At the TCDB, proteins are classified based on five criteria, and each of these criteria corresponds to one of the five numbers or letters within the Transporter Classification (TC) number for a particular type of transporter (e.g., 2.A.1.1.5, is the TC number for a hexose transporter from *S. cerevisiae*). Thus, as a result of BLAST search, a complete full Transportome of *S. indica* was obtained, formed by 764 proteins, all these related to transport to which a TC number was ascribed (Supplementary Table S2). The set of transporters of *S. indica* were grouped according to TCDB classification as shows in Figure 2. The most numerous classes of transporters are the Electrochemical potential-driven transporters (288 proteins), follows by the Primary active transporters (214) and the Channels/pores (107). Besides, other groups of proteins named “Accessory factors involved in transport” (77), “Incompletely characterized transport systems” (60), “Group translocators” (11) and “Transmembrane electron carriers” (7) were compiled (Figure 2). Interestingly, it should be noted that, according to the classification criteria of this database, the complete transportome includes proteins that cannot be considered as “true transporters.” In fact, the prediction of transmembrane (TM) domains of the *S. indica* proteins showed that, 347 out of the 764 proteins had 0 TMs, being some of them included in the last four groups of proteins mentioned above in which not true transporters but rather auxiliary proteins of true transporters are grouped. The absence of TMs in some of these proteins could also be due to incorrect annotation of the *S. indica* proteome, with some truncated transporters missing transmembrane fragments. Interestingly, one recent publication on the cloning and functional characterization of a TRK K^+^ transporter of *S. indica* supports this explanation (Conchillo et al., 2021). Comparison of the number of transporters identified in *S. indica* with those of other fungi revealed the great variety that exists among them in the distribution of the different types of transporters or channels (Table 1). Figure 3 shows a circular packing scheme of the 764 transporters grouped into the different classes of the TCDB. The largest class encompasses the Electrochemical potential driven transporters (39.1%) which include uniporters, symporters and antiporters for the uptake and efflux of nutrients and metabolites, respectively, that are in different cell membranes. The second main class of the Primary active transporters (28%) enclosed two important superfamilies: the P–P bond-hydrolysis driven transporters (ATPases, ABC transporters, etc.) and the oxidoreduction-driven transporters. Finally, the third main class of Channel/pores (14%) consisted of several superfamilies: the α-type channels (including ion channels and aquaporins), the β-barrel porins, the vesicle fusion pores, and the membrane-bounded channels (mainly the nuclear pore complex) (Table 1, Supplementary Table S2 and Figure 3).

**Table 1:** Distribution of the main classes of transporters in fungal transportome used in this study.

Class of transporters	*Serendipita indica*		*Saccharomyces cerevisiae*		*Cryptococcus neoformans*		*Aspergillus nidulans*	
	Transporters	%	Transporters	%	Transporters	%	Transporters	%
Electrochemical Potential-driven	288	37.7	225	66	330	74	570	80
Primary Active	214	28	92	27	79	17.8	107	12
Channels/Pores	107	14	21	6.2	29	6.5	28	4
Total	764	100	341	100	445	100	710	100

Information on fungal transporters other than *S. indica* was obtained from TransportDB (http://www.membranetransport.org/transportDB2/index.html).

#### *In silico* prediction of substrates transported of *Serendipita indica* transportome

**Figure 4:**
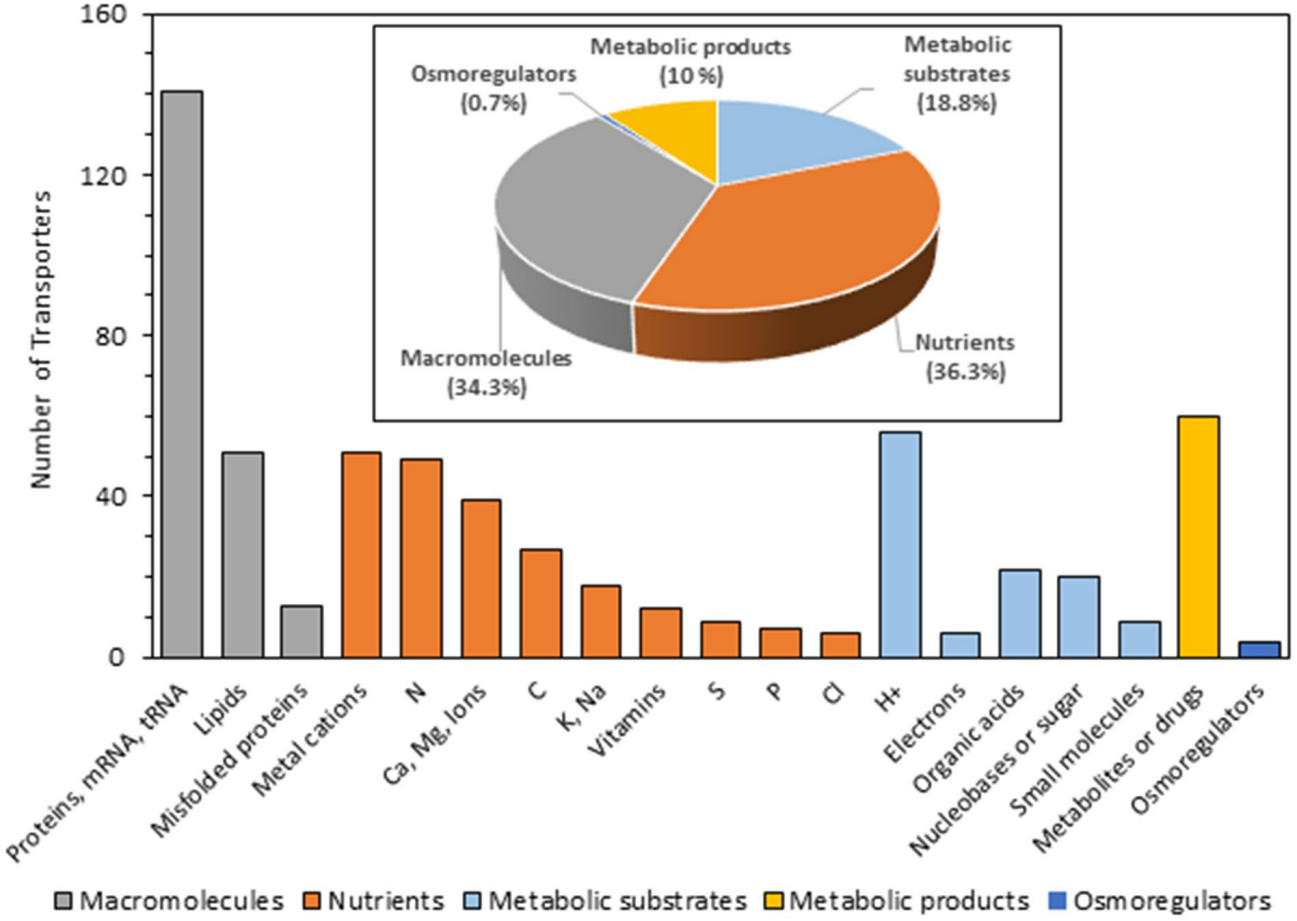
Transporters of *Serendipita indica* classified according to the putative substrate transported. A total of 607 transporters out of 764 has been studied. No information on the possible transported substrate has been found for the rest of the proteins (indicated in Supplementary Table S2 as “None” and “Unknown”). The bar graph represents the number of transporters grouped according to substrates, indicating with different colors those that transport macromolecules (gray), nutrients (brown), metabolic substrates (light blue), metabolic products (yellow) and osmoregulators (dark blue). This information was extracted by reviewing publications of homologous transporters from other organisms and from the TCDB where substrates are annotated based on the ChEBI ontology (https://www.ebi.ac.uk/chebi/) and by revising the literature.

To have functional information about the complete *S. indica* transportome, a classification of the 764 transporters was carried out on the basis of the transported substrate (Figure 4). For this purpose, the “Substrate Search Tool” available at TCDB was used to obtain information about the substrates of transporters homologous to those of *S. indica*.

Based on this *in silico* prediction, the final transportome obtained here revealed that the largest group of transporters and channels are involved in cellular fluxes of macromolecules such as proteins and lipids across mitochondrial, peroxisomal, RE or Golgi membranes as well as mRNA and tRNA fluxes though the nuclear pore complex to the cytoplasm (205 proteins) (Figure 4). In addition, *S. indica* has a plethora of transporters involved in macro and micro-nutrients supply and accumulation in cellular organelles (which together add up a total of 218 proteins, see Supplementary Table S2). Among them, the largest group of transporters is related to N nutrition, with a battery of 50 transporters and channels in charge of transporting amino acids (28), NH_4_^+^ channels (2), NH_4_^+^ transporters (2), urea (1), and oligopeptides (12) among others. Related to carbohydrate transport a total of 25 transporters were identified, most of them for monosaccharides (14), including high and low affinity for glucose among other monosaccharides; but also, for disaccharides (11). In addition, 8, 13, 10, and 12 homologs to fungal phosphate, potassium, sulfur, and vitamin transporters, respectively, were also identified. Besides the transporters that guarantee the supply of the main macronutrients, *S. indica* is endowed with several transporters for Ca^2+^, which are key players for Ca^2+^ homeostasis and signal generation. There are 21 between Ca^2+^ channels, Ca^2+^/H^+^ antiporters, and Ca^2+^-ATPases homologous to those present in other fungi. Regarding Mg^2+^, 8 channels and 1 electrochemical potential-driven transporter were identified and, interestingly, two of these Mg^2+^ transporters (PiMgT1, Protein ID_72581, and PiMgT2 Protein ID_72573) are among those not previously annotated in JGI that were included in the *S. indica* transportome in this study. The last pool of proteins grouped under the name of “metal cations” were those involved in the transport of various micronutrients such as Fe, Zn, Cu, Mn, and Ni, that together make up the largest group of nutrient transporters (66 transporters).

Other large cluster of transporters identified in the analysis of the transportome deals with efflux transporters responsible for removing substances that can be toxic to the cell (60, called “metabolic products” in Figure 4). This task is carried out by members of the ATP-binding cassette proteins (ABC proteins) or the Major Facilitator Superfamily (MFS). To avoid the detrimental effect of these toxic compounds, these proteins transport their substrates inside the vacuole or outside the cell.

Finally, an interesting group of transporters that may play a particularly relevant role when the fungus is exposed to stressful environmental conditions such as salinity are those related to Na^+^, K^+^, and Cl^−^ homeostasis as well as osmoregulatory transporters among which aquaporin and mechanosensitive channels are included. In *S. indica* transportome, a pool of 25 transporters have been identified that would be involved in Na^+^, K^+^, and Cl^−^ homeostasis which are compiled in Supplementary Table S3. The regulation in expression of some of them in response to salinity would suggest that they have a role that allows the fungus to withstand this stress condition (see below).

#### *In silico* prediction of subcellular location of *Serendipita indica* transportome

**Figure 5:**
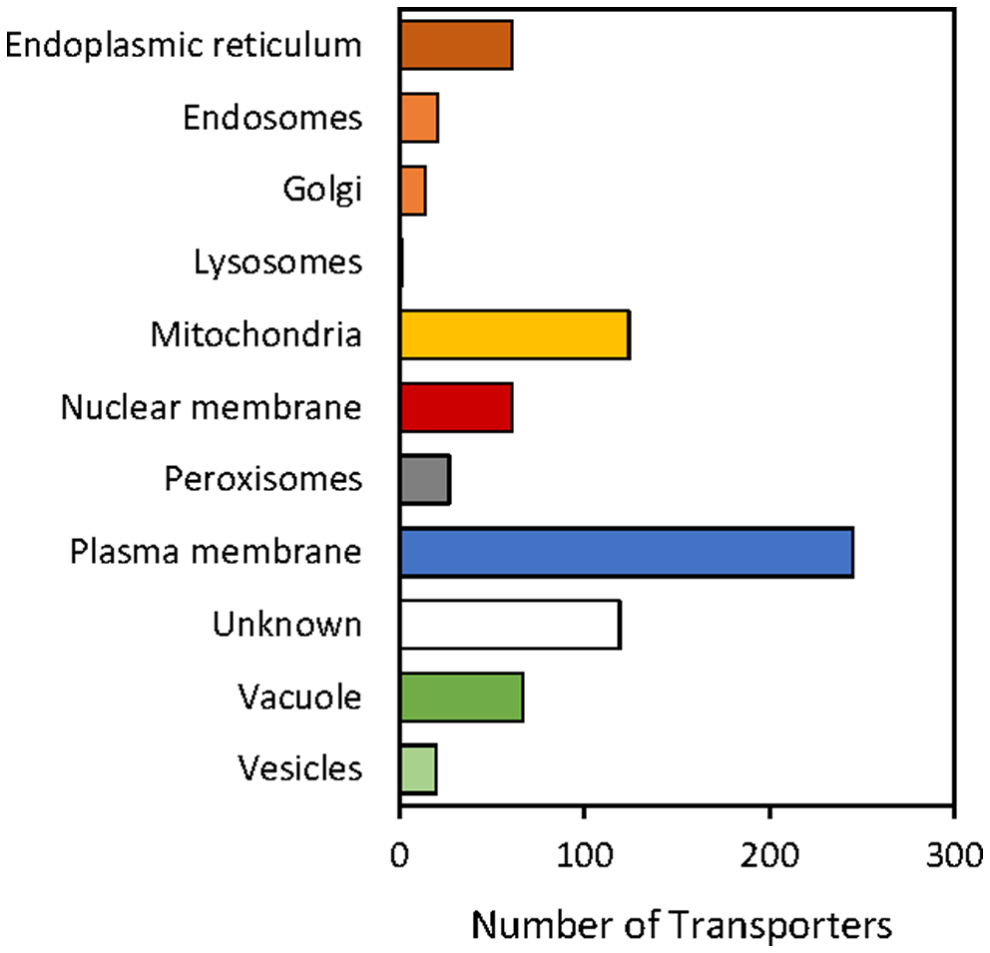
Transporters of *Serendipita indica* classified according to the subcellular location. A total of 643 transporters out of 764 has been studied. No information on the possible subcellular location has been found for the rest of the proteins (indicated in Supplementary Table S2 with “?”). This information was extracted from descriptions in the literature and TCDB annotations of homologous transporters in other organisms.

Regarding the putative subcellular location in which the *S. indica* transporters are active, a bar graph representation was also summarized in Figure 5. The proposed location for each transporter was deduced from information appearing in the TCDB or in the literature for homologous proteins in other organisms (see Supplementary Table S2). The results showed that the plasma membrane is where a greater number of transporters are located, followed by the mitochondrial and vacuolar membranes (Figure 5). This would suggest that the exchange with the external environment could be the transport activity to which the cell devotes more protein resources, even more than for ATP synthesis and cellular energization (carried out in the mitochondria) or to ensure reserves in the vacuole.

### Transcriptomic analysis of *Serendipita indica* in response to moderate saline conditions and under symbiosis

To adapt to high-Na^+^ environments, fungi have evolved multiple mechanisms for circumventing the toxic effects of Na^+^ which are substantially mediated by transporters that exclude Na^+^ from the cytosol. However, under these stressful conditions, some activity of other transport proteins responsible for fungal nutrition must also be maintained to ensure their survival. To find out which are the active actors of the *S. indica* transportome under saline conditions, a comprehensive transcriptomic analysis was carried out and the fungal genes expressed during growth under free-living conditions and during symbiosis with *Arabidopsis* were studied. The scheme of the experiment carried out is shown in Supplementary Figure S1.

#### Expression of the *Serendipita indica* transportome in response to salt in free living conditions

To know which genes encoding transporters were expressed in any of the growth conditions tested, FPKM corresponding to any of the 764 genes that make up the transportome were analyzed. It was found that 12 out of 764 *S. indica* transporters were not expressed in some of the studied conditions (Supplementary Table S4).

**Figure 6:**
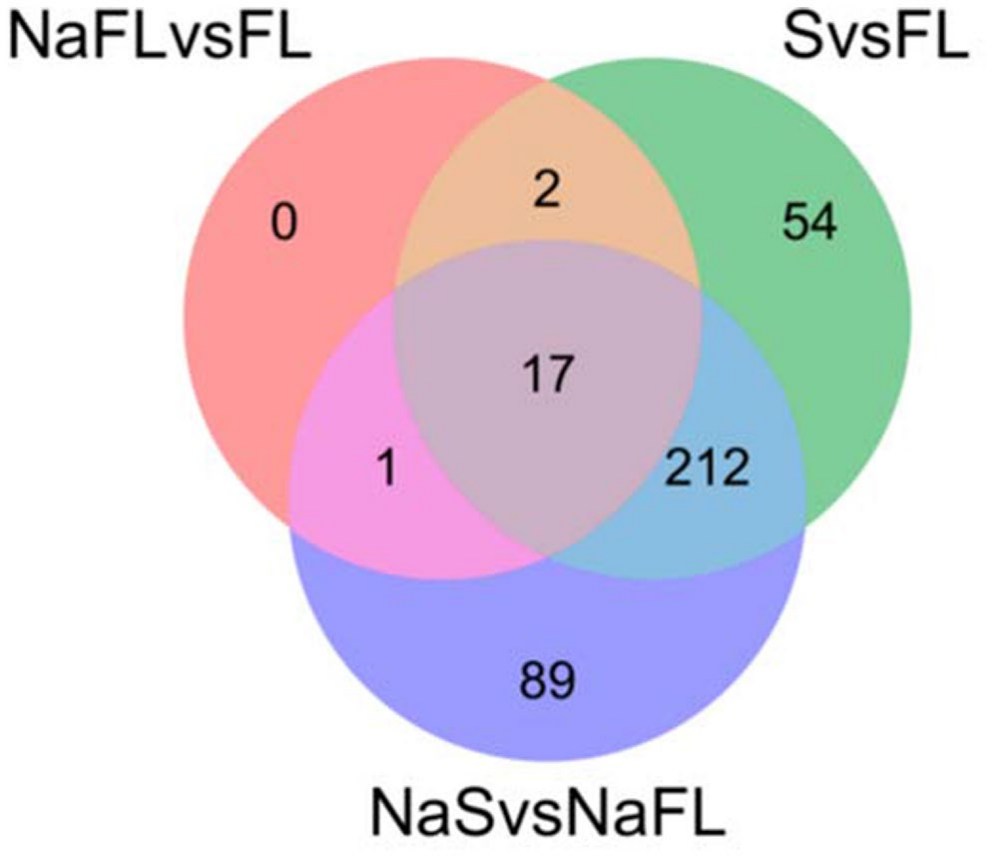
Representation of Venn diagram of the comparisons between differential expressed genes (DEGs) encoding transporters NaFL vs. FL; FL vs. Symb and NaFL vs. NaSymb. It shows that the 20 DEGs of transporters that were identified in response to salt in free living conditions (NaFL vs. FL) were also differentially expressed in response to symbiosis (FL vs. S) and/or in response to salinity during symbiosis (NaS vs. NaFL). This representation was obtained using Venny interactive tool (https://bioinfogp.cnb.csic.es/tools/venny/).

To determine the transcriptional response of *S. indica* to salinity, differentially expressed genes (DEGs) encoding transporters were compared between the fungal samples grown under control conditions and after the salt treatment at the same time of incubation, either in free living conditions (the comparisons of DEGs NaFL vs. FL: FL1.5 h vs. FLNa1.5 h; FL7d vs. FLNa7d; FL14d vs. FLNa14d) or under symbiosis (the comparisons of DEGs NaSymb vs. Symb: Symb1.5 h vs. SymbNa1.5 h; Symb7d vs. SymbNa7d; Symb14d vs. SymbNa14d). Of the 752 genes encoding transporters for which some FPKM value was obtained, a total of 375 genes were differentially expressed in some conditions tested. As shown in Table 2, few changes in gene expression were observed in response to salinity under free living conditions (346 total DEGs) and there were no changes at all in response to salt during symbiosis. Under free living conditions, of the 346 DEGs, 20 genes encoding transporters were differentially expressed, with 11, 6 and 6 DEGs retrieved after 1,5 h, 7, or 14 days, respectively (Table 2). Interestingly, the Venn diagram representation of DEGs of transporters in the comparisons between NaFL vs. FL; FL vs. Symb, and NaFL vs. NaSymb showed that none of the 20 transporters were regulated exclusively in response to salt but also in the switch to symbiosis (Figure 6). Of the 20, three transporters were related to Na^+^, K^+^, and Ca^2+^ homeostasis and among them, the *SiENA5* gene (corresponding to Protein ID: PIRIN_72634), which encodes a Na^+^ efflux ATPase from *S. indica* (Lanza et al., 2019) is noteworthy. It was the only DEG that was consistently upregulated in saline conditions at the three times of the experiment studied (Table 3). Besides, *SiKHA1* (Protein ID: PIRIN_75414), a gene which encodes a Golgi localized K^+^-H^+^ antiporter (Maresova and Sychrova, 2005; Conchillo et al., 2021), was also induced upon salinity while a gene homologous to vacuolar CAX-type Ca^2+^, H^+^ exchanger (Protein ID: PIRIN_72312) was significantly repressed (see Table 3).

**Table 2:** Transporters of *Serendipita indica* differentially expressed in two comparisons: studying the response to salinity and to the lifestyle change toward symbiosis.

Growth conditions compared in this study	Total DEGs	DEGs of transporters	DEGs at 1.5 h		DEGs after 7 days		DEGs after 14 days	
			Up	Down	Up	Down	Up	Down
NaFL vs. FL	346	20	6	5	6	0	5	1
NaSymb vs. Symb	9	0	–	–	–	–	–	–
Symb vs. FL	3,548	285	75	30	89	71	102	77
NaSymb vs. NaFL	4,074	319	79	64	89	88	93	100

**Table 3:** DEG of transporters of *Serendipita indica* regulated by salt in free living conditions.

Protein_id	PIIN	Family*	Putative substrate	Subcellular location**	Fold change (log2 ratio)		
					NaFL1.5 h/FL1.5 h	NaFL7d/FL7d	NaFL14d/FL14d
72634 (SiENA5)	PIIN_01840	P-ATPase	K^+^, Na^+^	PM	1.94	1.39	1.64
75414 (SiKHA1)	PIIN_04607	CPA2	K^+^, H^+^	ER, Golgi	1.04	0	0
76836	PIIN_06030	MFS	Metabolites	PM	1.49	0	0
80618	PIIN_09801	MFS	Metabolites	PM	1.41	0	0
79280	PIIN_08471	MFS	Metabolites	PM	1.27	0	1.34
76839	PIIN_06033	MFS	Metabolites	PM	0	0	1.47
73068	PIIN_02271	OPT	Oligopeptides	ER	0	0	1.14
77505	PIIN_06701	MFS	Vitamins	PM	0	0	1.15
72591	PIIN_01799	MFS	Glucose	PM	1.59	0	0
75164	PIIN_04361	MFS	Xylose	PM	0	1.05	0
75943	PIIN_05137	MFS	Galactose, H^+^	PM	0	1.41	0
71636	PIIN_00866	PiT	Phosphate, Na^+^	PM	0	1.5	0
72809	PIIN_02015	Triflin or CRISP	None	?	0	1.51	0
79846	PIIN_09033	DMT	Unknown	?	0	1.07	0
72312	PIIN_01532	CaCA	Ca^2+^, H^+^	Vacuole	−2.96	0	0
78458	PIIN_07651	Calmodulin	Phospholipids	?	−1.88	0	0
74378	PIIN_03575	NPC	Phospholipids	Nucleus	−1.14	0	0
73489	PIIN_02689	NDH	H^+^	Mitochondria	−1.06	0	0
72519	PIIN_01732	QCR	H^+^	Mitochondria	−1.09	0	0
75212	PIIN_04408	NDH	H^+^	Mitochondria	0	0	−1.02

*CPA2, Cation-proton antiporter; MFS, Major Facilitator Superfamily; OPT, Oligopeptide Transporter; DMT, Drug/Metabolite Transporter; CaCA, Ca^2+^:Cation Antiporter; NPC, Nuclear Pore Complex; NDH, H^+^ or Na^+^-translocating NADH Dehydrogenase; QCR, Proton-translocating Quinol: Cytochrome c Reductase.

**PM, Plasma membrane.

Cells colored in red mean upregulation and blue mean downregulation.

To find out if any of the transporters regulated in response to salt that do not belong to the list of those related to Na^+^, K^+^, and Cl^−^ homeostasis can play any role in mitigating the Na^+^ effect on fungal cells, those expressed in plasma membrane were filtered out. They were 10 transporters and, interestingly, all of them were induced in response to NaCl at any time of the experiment (Table 3). Among them, some metabolite efflux transporters belonging to the major facilitator superfamily (MFS) were included (Protein ID: PIRIN_76836, PIRIN_76839, PIRIN_79280, and PIRIN_80618). In addition, 3 DEGs encoding sugar transporters (Protein ID: PIRIN_72591, PIRIN_75164, and PIRIN_75943) were induced under saline conditions in a similar manner to that described for *HXT1* from *S. cerevisiae* (Hirayama et al., 1995); and a gene encoding a phosphate transporter homologous to the Na^+^-symporter PHO89 from *S. cerevisiae* (Protein ID: PIRIN_71636), as well as a nicotinate transporter gene (Protein ID: PIRIN_77505) were also induced in response to NaCl (see Table 3).

Among the repressed genes, it is worth highlighting three genes encoding transporters related to mitochondrial respiratory functions (Protein ID: PIRIN_72519, PIRIN_73489, and PIRIN_75212) what indicates that a metabolic failure of the fungus can be occurred in response to salinity.

#### Expression of the *Serendipita indica* transportome in response to salt and symbiosis

To know which transporters from *S. indica* may have a role during the establishing and maintenance of the symbiotic relationship with *Arabidopsis* roots, a differential expression analysis was performed between free living conditions and during co-cultivation with plants growing in the absence and presence of 50 mM NaCl. Interestingly, only 9 total DEGs were identified at any time of the experiment in comparison of DEGs NaSymb vs. Symb and none of them were a gene encoding transporter (Table 2).

**Figure 7:**
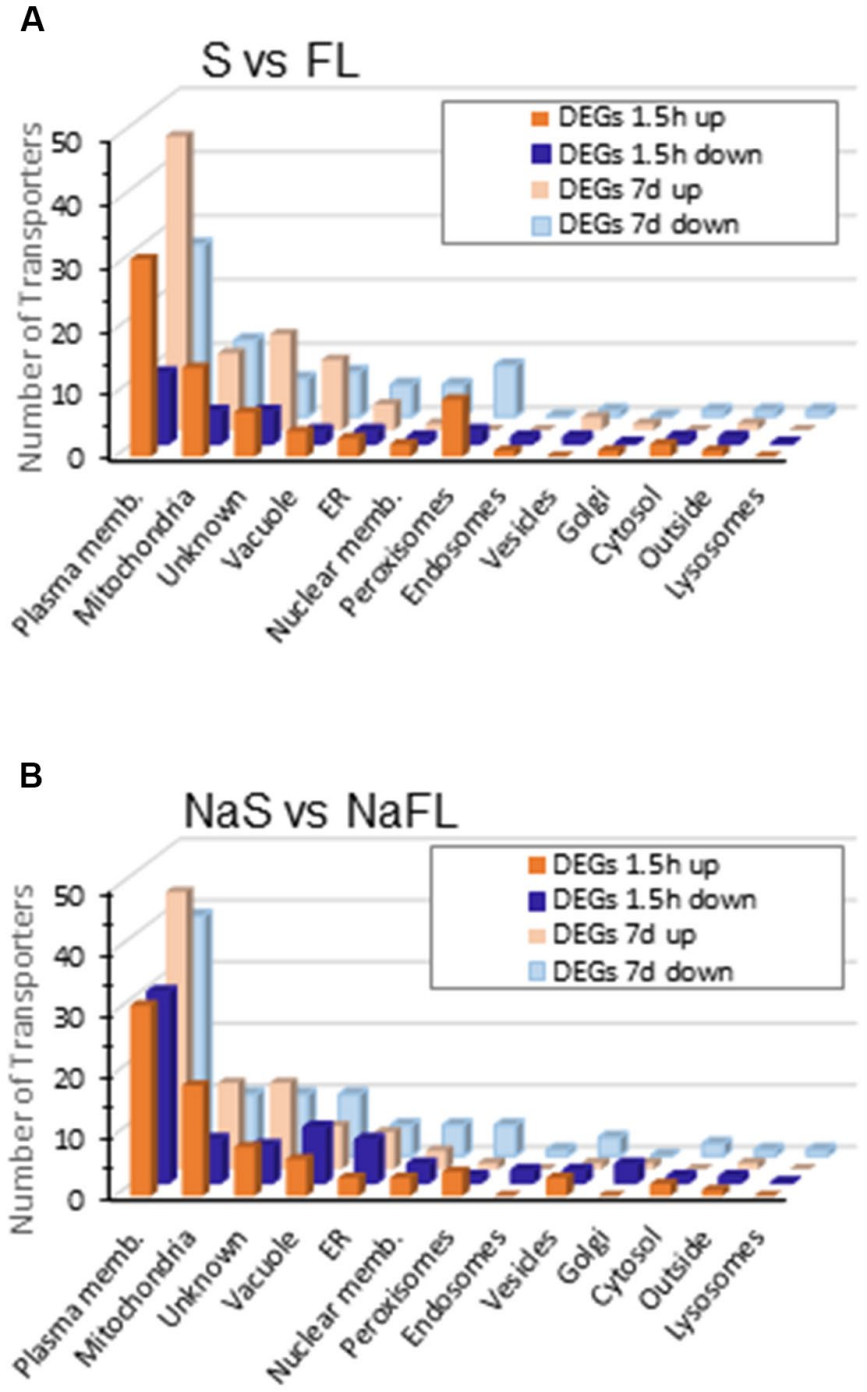
Differentially expressed genes encoding transporters during symbiosis at 1.5 h and 7 days of colonization grouped according to cellular location. The number of transporters grouped according to subcellular location that are regulated between free living and symbiosis **(A)** or free living and symbiosis in the presence of 50 mM NaCl **(B)** is represented in a bar graph. In yellow are represented the DEGs that are induced at 1.5 h (intense yellow) or after 7 days of colonization (pale yellow); in blue are represented the DEGs that are repressed at 1.5 h (intense blue) or after 7 days of colonization (pale blue).

Opposite to this low response to salinity, a large amount of total DEGs were identified in the change of growth from free style life to symbiosis identifying 3.548 and 4.074 total DEGs in the Symb vs. FL and NaSymb vs. NaFL comparisons, respectively (Table 2). Of these, a total of 375 DEGs encoding transporters were detected, 285 DEGs during the fungal growth in symbiosis in the absence of NaCl and 319 DEGs in the presence of NaCl (Table 2 and Supplementary Table S5). The distribution of these transporters among the cellular membranes showed that the main compartment actively regulated upon *S. indica* infection was the plasma membrane where many transporters were differentially induced (Figure 7). Strikingly, in the early stages of infection (1.5 hpi) in the absence of NaCl, peroxisomal proteins were upregulated, whereas when symbiosis was established (7–14 dpi), the number of peroxisomal responsive proteins decreased and those that were regulated showed a global repression (Figure 7A). Under saline conditions, the upregulation of peroxisome transporters in the initial stages of the infection was not evident (Figure 7B).

**Figure 8:**
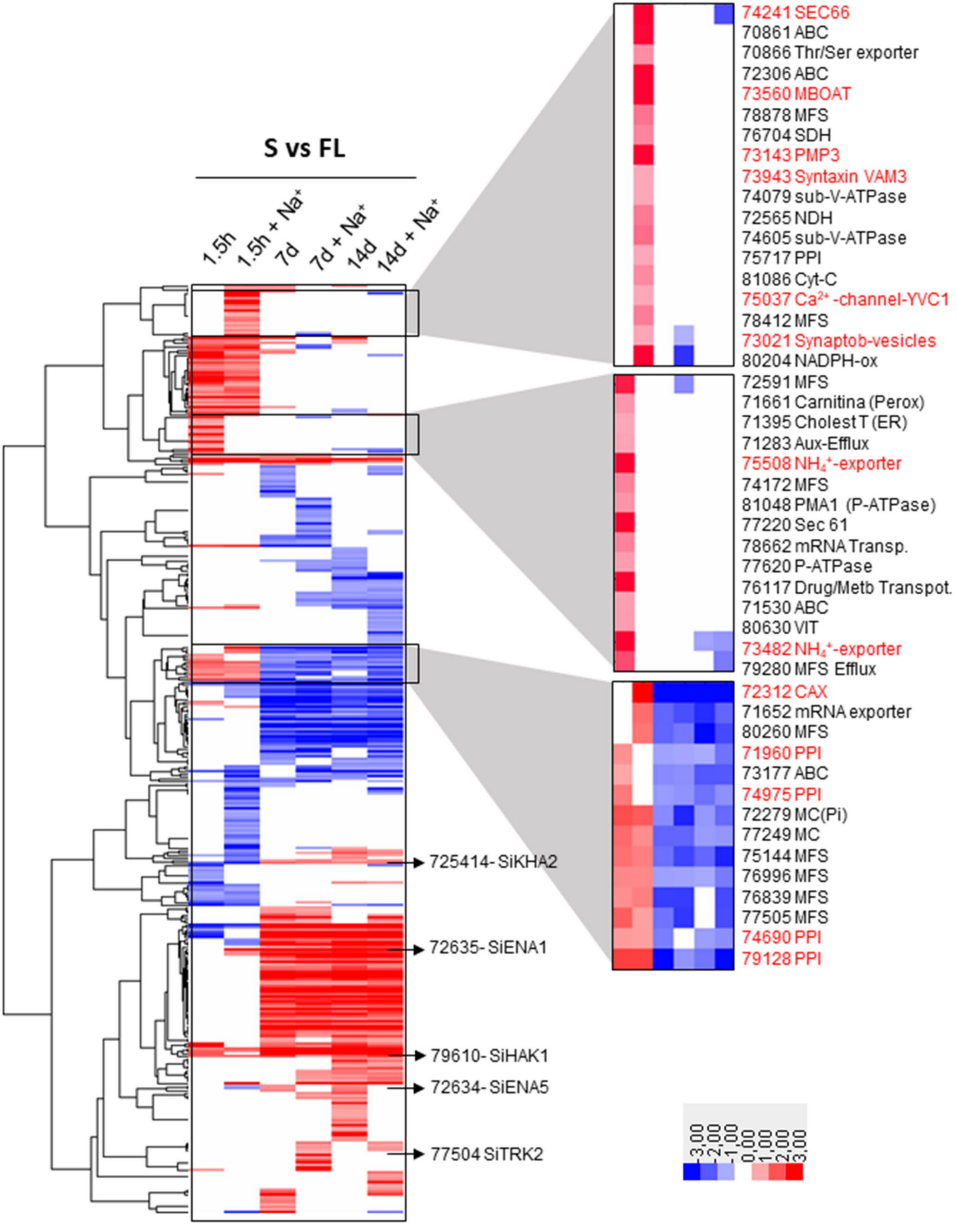
Hierarchical clustering heatmap of DEGs of transporters regulated in symbiosis. The 375 DEGs of transporters that are regulated in symbiosis are represented indicating the level of expression with a color code (red, upregulated; blue, downregulated). Several individual genes related to Na^+^ and K^+^ homeostasis that are induced in any of the comparison tested are highlighted. In addition, three sets of co-regulated transporters are also highlighted. Among them are those that were induced exclusively 1.5 hpi in the presence of NaCl (1.5 h + Na^+^); genes that were exclusively induced 1.5 hpi in the absence of NaCl (1.5 h); and genes that were induced at the beginning of colonization but were repressed at long colonization times (7 d, 7 d + Na^+^, 14 d, and 14 d + Na^+^). The Protein ID number of the genes mentioned in the text are highlighted in red. The rest are written in black naming the family of transporters to which they belong. ABC, ATP Binding Cassette family; MBOAT, membrane-bound O-acyltransferase; MFS, Major Facilitator Superfamily; SDH, Succinate Dehydrogenase; PPI, Peroxisomal Protein Importer; NDH, NADPH Oxidase; VIT, Vacuolar Iron Transporter; MC, Mitochondrial Carrier.

The hierarchical clustering of total 375 DEGs encoding transporters showed different patterns of expression (Figure 8 and Supplementary Table S5), among which the following are worth mentioning: (i) very few genes that were induced at all times and conditions under symbiosis including the gene encoding the high-affinity K^+^ uptake system *SiHAK1* (Protein ID_79610) that can be highlighted; (ii) group of few DEGs that responded by induction or repression to salinity in early infection phase at 1.5 hpi; (iii) groups of DEGs that were clearly distinguished according to the time of colonization but independently of the saline treatment: on the one hand there is a group of those that were regulated in the early stages of infection (1.5 hpi) and, on the other hand, those that were regulated in late stages of symbiosis (7 and 14 dpi), without observing notable differences between these two long times studied (Figure 8 and Supplementary Table S5). Among the DEGs that were induced exclusively in response to salt at 1.5 hpi, no transporter related to Na^+^, K^+^, and Cl^−^ homeostasis was included. However, 3 genes encoding proteins of the vesicle secretion system were in this group (Protein ID_74241, a homolog to Sec66; Protein ID_73021, a Synaptobrevin homolog; and Protein ID_73943, Syntaxin VAM3), being markedly induced Sec66 which reached a fold change of 11.8, the highest level on induction among all. Interestingly, their induction would support the involvement of the vesicle secretion system in the movement of NaCl in cells previously described in plants, which would exist as a parallel route for the transport of Na^+^ from the outside to be discharged into the vacuole by endocytosis and, in turn, could remove Na^+^ from the cell to the outside by exocytosis (Leshem et al., 2006; Baral et al., 2015; Garcia de la Garma et al., 2015; Flowers et al., 2019; Orlova et al., 2019). Another membrane protein that was significantly induced in response to salt was Protein ID_73143, an homolog to PMP3 of *S. cerevisiae* and RCI2A from plants, described as a regulator of membrane potential under saline conditions (Navarre and Goffeau, 2000; Nylander et al., 2001). One more DEG that was induced under saline conditions in symbiosis was Protein ID_73560, encoding a homolog of the yeast Gup1, initially described as glycerol transporter that now has been demonstrated to be an membrane-bound O-acyltransferase (MBOAT) involved in fundamental biological processes such as polarity establishment, secretory/endocytic pathway functionality, vacuole morphology and cell wall and membrane composition (Lucas et al., 2016). In previous work, the yeast Gup1/HHATL encoding gene has shown to be induced upon NaCl shock (Yale and Bohnert, 2001). Finally there were two DEGs encoding vacuolar Ca^2+^ transporters that were highly regulated under saline conditions, one that is homologous to *S. cerevisiae* YVC1/TrpY1 (Protein ID_75,037), and is activated by stretch to release vacuolar Ca^2+^ into the cytoplasm upon osmotic shock (Zhou et al., 2005); and the other a CAX-type vacuolar transporter (Protein ID_72,312), whose rice homologs are induced by salt stress (Yamada et al., 2014). Regarding those genes that were induced at early colonization stage (1.5 hpi) in symbiosis with respect to free life regardless salinity, there were 37 DEGs among which seven metabolite efflux systems that can be involved in extrusion of fungal effectors (Figure 8 and Supplementary Table S5). It is also worth mentioning a small group of genes that are induced in early stages of infection and repressed in later stages. Curiously, three metabolite or drug efflux systems and four peroxisomal transporters are also found in this group (Figure 8 and Supplementary Table S5).

At late stages of colonization, there was a group of numerous DEGs significantly induced or repressed under symbiosis (Figure 8 and Supplementary Table S5), regardless the saline treatment without remarkable changes in expression between 7 and 14 dpi. A set of 51 genes was identified that were exclusively induced after 7 and 14 dpi, most of them being nutrient or metabolite transporters. Interestingly, this group included *SiENA1* (Protein ID_72635) and *SiKHA2* (protein ID_75414), both related to Na^+^ and K^+^ homeostasis, the former being one of the DEGs that was the most induced during symbiosis (Figure 8 and Supplementary Table S5).

The classification of the 375 transporter DEGs regulated under symbiosis according to the substrate transported showed that there were numerous DEGs of transporters involved in the macromolecule fluxes (proteins, diverse RNA molecules and lipids) that are actively regulated under symbiosis (Supplementary Table S5). In addition, representative genes involved in the transport of the major macronutrients (C, N, P, K, S) and micronutrients were also significantly regulated; some were induced, and others were repressed at the early or late stages of infection, indicating that *S. indica* has a wide repertoire of transporters that are specifically active in the different fungal lifestyles and growth conditions to ensure its adequate nutrition (Supplementary Table S5).

Among the transporters of macronutrients, those related to N nutrition, which form a numerous and diverse group (including transporters of oligopeptides, amino acids, ammonium, and urea) deserve especial attention. There are 28 DEGs that were regulated in some of the conditions of symbiosis (Supplementary Table S5). As shown in Supplementary Table S5, a compact group of DEGs were only induced in the late stages of symbiosis, among which some were not regulated or repressed in the early stages of colonization. Examples of those repressed at 1.5 hpi and induced after 7 and 14 dpi are two transporters involved in NH_4_^+^ (Protein ID_72831 and Protein ID_75176) and one in urea uptake, the latter homologous to DUR3 (Protein ID_74652). In contrast, two DEGs encoding NH_4_^+^ exporters (Protein ID_73482 and Protein ID_75508) were highly induced only in the early stages of colonization.

#### Regulation of genes involved in ionic homeostasis in salinity and symbiosis

**Figure 9:**
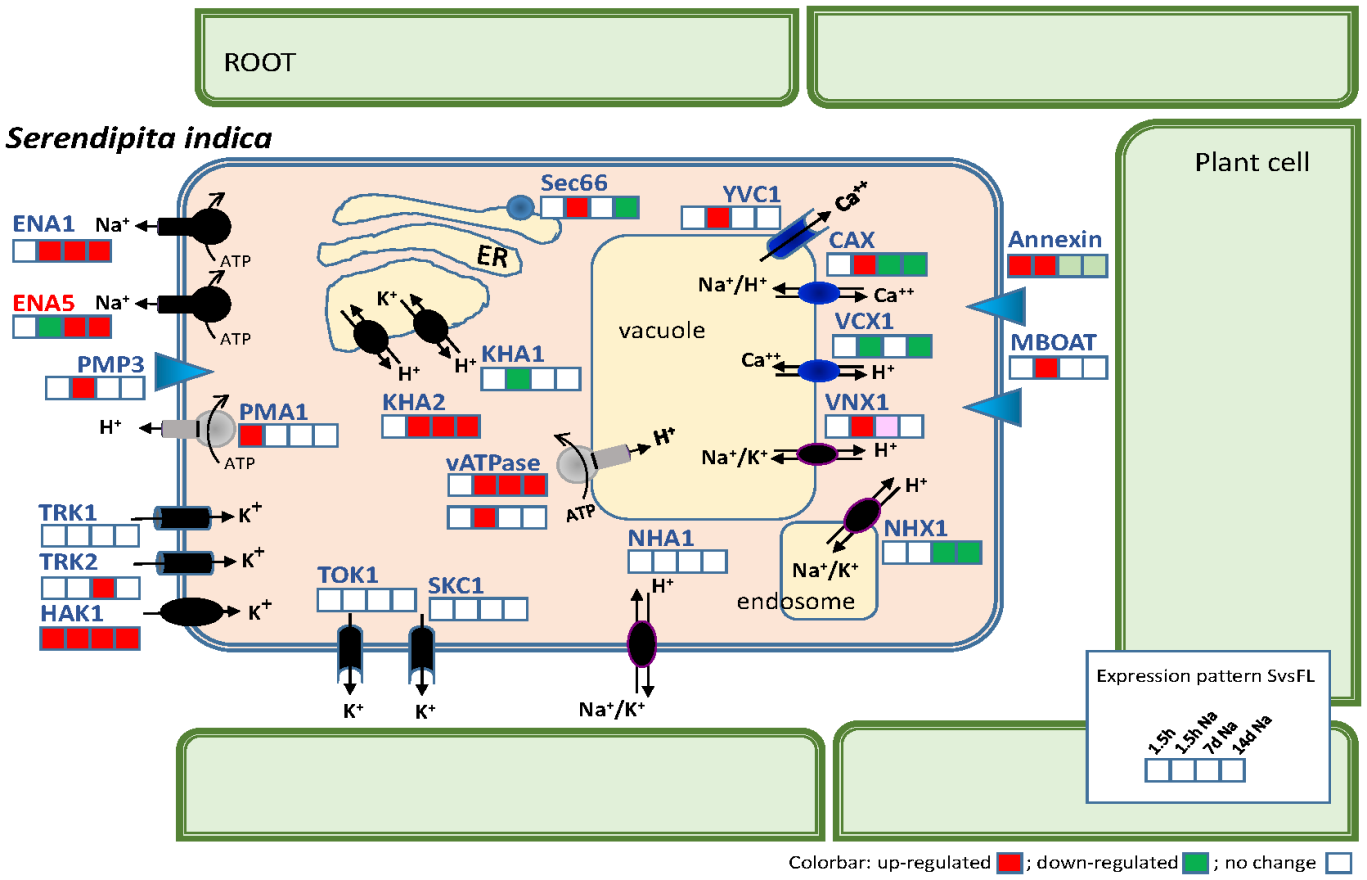
Scheme summarizing the transporters related to Na^+^, K^+^, H^+^, and Ca^2+^ homeostasis of *Serendipita indica* and their gene expression levels during symbiosis with *Arabidopsis*. For each gene, the gene response is indicated in four colored squares according to the comparisons between Symb vs. FL after 1.5 h of infection; after 1.5 h of infection in the presence of 50 mM NaCl; 7 days of infection with NaCl; and 14 days of infection with NaCl. At long colonization times (7 and 14 days) no striking differences were observed between control and NaCl conditions and therefore only one condition is represented. The color code is red, upregulated; green, downregulated; and white, no change in expression.

To find any evidence about which transporters may be involved in maintaining the Na^+^ and K^+^ homeostasis during the growth of the fungus in symbiosis, the analysis of DEGs of this group of transporters (Supplementary Table S3) was performed. In addition, we studied the expression of transporters involved in H^+^ fluxes that, directly or indirectly, can regulate the cytoplasmic pH and the H^+^ gradient across membranes on which other transporters depend for their functioning. Furthermore, Ca^2+^ transporters were also analyzed, since cytoplasmic Ca^2+^ levels can influence cellular Na^+^ and K^+^ homeostasis (Lynch et al., 1989; Li et al., 2006). As shown in Table 4, a total of 22 genes encoding transporters were regulated in some of the studied comparisons. Of these, the most relevant transporters are compiled in Figure 9 together with a plot of gene expression responses at the different times of the experiment.

**Table 4:** Relevant transporters of *Serendipita indica* for the ionic homeostasis in moderate salinity.

Protein	Fold change (log2)						Family	Substrates	Complementary description	Cellular location
	1.5 hS/FL	1.5 hNaS/NaFL	7dS/FL	7dNaS/NaFL	14dS/FL	14dNaS/NaFL				
81048	1.24	0	0	0	0	0	P-type ATPase (P-ATPase) Superfamily	H^+^	SiPMA1.H^+^-ATPase	PM
71428	0	0	1.22	1.33	1.31	1.69	F-type, V-type and A-type ATPase (F-ATPase) Superfamily	H^+^	Vacuolar ATPase subunit	Vacuole
74605	0	1.70	0	0	0	0	F-type, V-type and A-type ATPase (F-ATPase) Superfamily	H^+^	Vacuolar ATPase subunit D	Vacuole
79610	1.85	1.88	2.48	1.04	1.99	2.55	K^+^ Uptake Permease (KUP) Family	K^+^	SiHAK1. K^+^ Uptake Permease (HAK)	PM
75409	0	−1.30	0	0	0	0	Monovalent Cation:Proton Antiporter-2 (CPA2) Family	K^+^, H^+^	SiKHA1. Potassium-proton antiport	ER, Golgi
75414	0	−1.69	1.09	1.50	1.06	1.36	Monovalent Cation:Proton Antiporter-2 (CPA2) Family	K^+^, H^+^	SiKHA2. Potassium-proton antiport	ER, Golgi
72635	0	3.120	7.80	9.77	10.50	11.41	P-type ATPase (P-ATPase) Superfamily	K^+^, Na^+^	SiENA1, K^+^, Na^+^ efflux system	PM
72634	0	−1.37	1.27	0	1.52	0	P-type ATPase (P-ATPase) Superfamily	K^+^, Na^+^	SiENA5, K^+^, Na^+^ efflux system	PM
77504	0	0	0	1.09	0	0	K^+^ Transporter (Trk) Family	K^+^, Na^+^	SiTRK2 (partial). K^+^ uptake	PM
72636	0	1.06	1.08	0	0	0	Monovalent Cation:Proton Antiporter-2 (CPA2) Family	Na^+^, H^+^	SiVNX1. Na^+^/H^+^ antiporter from the cytoplasm into the thylakoid lumen	Mitochond
74006	0	0	0	−1.40	−1.26	−1.08	Monovalent Cation:Proton Antiporter-1 (CPA1) Family	Na^+^, H^+^	SiNHX1. exchange of protons for cations such as Na^+^ or K^+^	Vacuole
75037	0	1.01	0	0	0	0	Transient Receptor Potential Ca2^+^ Channel (TRP-CC) Family	Ca^2+^	YVC1. Vacuolar, voltage-dependent cation-selective, Ca^2+^-activated channel	Vacuole
71526	2.03	2.27	0	−1.80	0	0	α-Type Channels	Ca^2+^	Annexin D1	
74614	0	−2.45	0	0	0	−1.75	Ca^2+^:Cation Antiporter (CaCA) Family	Ca^2+^, H^+^	VCX1, has a role in intracellular calcium ion sequestration	Vacuole
72312	0	7.86	−5.89	−5.02	−6.10	−5.72	Ca^2+^:Cation Antiporter (CaCA) Family	Ca^2+^, H^+^	CAX, The vacuolar Ca^2+^:H^+^ exchanger	Vacuole

Cells colored in red mean upregulation and blue mean downregulation.

Among the transporters mediating H^+^ fluxes, the gene encoding the plasma membrane H^+^-P-ATPase of *S. indica*, *SiPMA1* (Protein ID_81048) showed an induction at the beginning of infection (1.5 hpi) in the absence of Na^+^ (Table 4). This early induction was like that described in other symbiotic fungi such as *Uromyces* (Struck et al., 1998), which could be required at the initiation of symbiotic growth, probably to provide sufficient capacity for active nutrient transport. Besides, a recently described Hv-like H^+^ channel in fungi (Zhao and Tombola, 2021), was also induced after 7 dpi in the presence of NaCl (Protein ID_72827). In addition, two DEGs encoding subunits of V-ATPases were also induced, one of them in presence of NaCl at early infection stage (1.5 hpi) (Protein ID_74605) and the other one during the late symbiosis with and without NaCl (Protein ID_71428). Regarding Ca^2+^ fluxes, as above mentioned, two vacuolar Ca^2+^ transporters were induced by NaCl at 1.5 hpi. These results could point to vacuoles as storage organelles during a stress situation, to accumulate Na^+^ during the saline growth or to regulate cytoplasmic Ca^2+^ levels as second messenger triggered in response to salinity. In reference to the salt-induced activation of cytoplasmic Ca^2+^ fluxes, interestingly, the induction of *S. indica* homologous annexin (Protein ID_71526), was also observed, in early stages of colonization (Table 4). Annexins are proteins associated with the plasma membrane that in plants mediate the entry of Ca^2+^ into the cytoplasm in response to salinity (Laohavisit et al., 2013).

Analyzing those transporters related to Na^+^ and K^+^ homeostasis in other fungi, no differential expression of the *SiNHA1* gene was detected in any of the comparisons and, interestingly, *SiNHX1* was significantly repressed after 7 and 14 dpi, both in the absence (14 dpi) and presence of NaCl (7 and 14 dpi) (Table 4). These results support the previously stated idea that both genes should have no relevant role in the fungal response to salt or to symbiotic life. The gene expression of other *S. indica* transporters such as *SiKHA1* and *SiKHA2* and *SiVNX1* found in inner membranes such as the ER, Golgi or mitochondria was also significantly regulated as shown in Table 4, standing out *SiKHA2*, which was specifically upregulated after 7 and 14 dpi, regardless the presence of NaCl, which could denote a notable role in internal ionic homeostasis. Probably SiKHA2 contribution would be the accumulation of Na^+^ and/or K^+^ in the organelles to balance a proper ionic strength of the cytosol when the symbiosis was established.

As mentioned above, the gene encoding the high-affinity K^+^ uptake system *SiHAK1* (Protein ID_79610) was induced in all the symbiosis conditions studied (in the presence and absence of NaCl) with respect to free living conditions. This suggest that it could be the main transporter in charge of the K^+^ nutrition of the fungus during the symbiosis. However, it should be noted that another *S. indica* K^+^ uptake system, *SiTRK2*, was also induced but exclusively at some point during the symbiosis under saline conditions.

**Figure 10:**
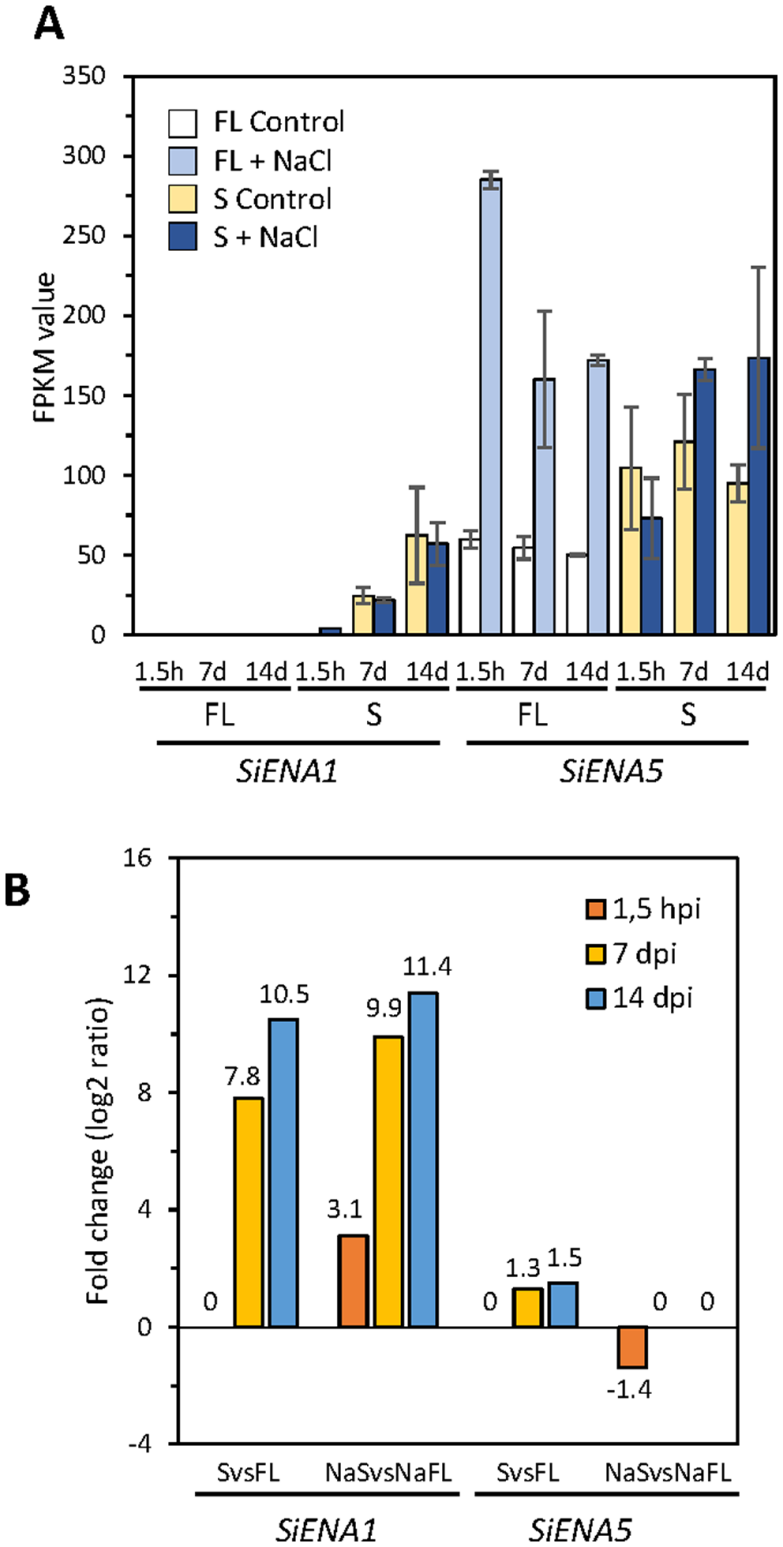
Expression of *ENA* genes of *Serendipita indica* in different growth conditions. In **(A)** the expression of the *ENA* genes *SiENA1* and *SiENA5* in FPKMs is represented for each of the free-living conditions and symbiosis studied (with and without NaCl) and at the different culture times, 1.5 h, 7 and 14 days. In **(B)** the differential expression of the *ENA* genes is represented in the comparisons between free life and symbiosis in the absence (FL vs. Symb) and in the presence of NaCl (NaFL vs. NaSymb) at the different incubation times.

On the other hand, the *SiENA1* gene (Protein ID_72635) was induced in the presence of NaCl at beginning of infection (1.5 h dpi) and during the symbiosis independently of Na^+^, being surprisingly the gene that showed the highest induction of all the transporters (Figure 10 and Table 4, log2ratio = 7.80 after 7d; and log2ratio = 10.51 after 14 days in the absence of NaCl). In parallel to *SiENA1*, a DEG encoding a Na^+^-phosphate transporter homolog to *ScPHO89* from *S. cerevisiae* (Protein ID_71636), was also strongly induced at 7 and 14 dpi during symbiosis resembling the coordinated induction in expression described previously of the two homologs transporter genes from *S. cerevisiae, ScENA1* and *ScPHO89* in response to an alkaline environment (Serra-Cardona et al., 2014).

Further analysis of the differential expression of the *S. indica ENA* genes showed that in terms of total expression (FPKM), *SiENA5* is expressed more than *SiENA1* in both free-living and symbiosis conditions regardless of the presence or absence of salt (Figure 10). However, the differential expression of *SiENA1* between FL vs. Symb and NaFL vs. NaSymb was markedly higher than that of *SiENA5* (Figure 10), indicating that the major role of SiENA1 might be associated with symbiosis, regardless of whether it is established under salinity conditions.

## Discussion

### SiNHA1 and SiNHX1, two Na^+^ transporters from *Serendipita indica*

One aspect addressed in this report has been the study of the mechanism/s by which the co-cultivation of the endophyte *S. indica* reduces the Na^+^ accumulation of plants under saline conditions. First, we focused on the possible contribution of *S. indica* Na^+^ transporters other than ENA ATPases that participate in Na^+^ homeostasis in other fungi such as NHA1 and NHX1 transporters. We have cloned and functionally characterized the homologous genes *SiNHA1* and *SiNHX1* of *S. indica* in Na^+^ defective yeast mutants. Here, it was shown that they complemented the mutant defect to grow in the presence of moderate NaCl concentrations only at low pH, conditions at which the H^+^ gradient is more favorable for its function (Figure 1). Regarding their gene expression and similar to what has been previously described for *NHA1* of *S. cerevisiae* (Ariño et al., 2018), they showed a transcriptional expression not inducible by salinity (Supplementary Table S4), both when the fungus lives under free conditions or in symbiosis, which does not rule out that their function may be post-translationally regulated under saline conditions.

To gain insight into other transport systems that may be involved in the regulation of cellular Na^+^ accumulation, we have extended the study to the entire *S. indica* transportome. For that, a comprehensive transcriptomic analysis was carried out by RNA-seq, with the idea of identifying transporters regulated by NaCl during free life and under symbiosis that could transport Na^+^. Before this, an exhaustive compilation of the proteins that make up the *S. indica* transporter set was approached as a prior step to identify candidates.

### A redefined transportome of *Serendipita indica* and its transcriptional expression in the transition to symbiosis

From the transporter data collected in JGI (Zuccaro et al., 2011), we have expanded the inventory of fungal transporters using two complementary strategies: by phylogenetic comparisons with transporters from other fungi and by analysis based on functional annotations using the eggNOG database. The combination of both strategies has proven to be very efficient in identifying other transporters not included in the JGI annotations. From 684 initially annotated proteins, we have increased the inventory to 764 proteins that may be involved in transport processes (Supplementary Table S2). The verification of their “transporter character” was confirmed by blasting all the new proteins against the TCDB, providing them with the corresponding TC number (from “Transport Classification number”). It is worth noting that, reviewing the length of proteins, the number of transmembrane fragments, and the functional annotations of the translated proteins annotated initially in JGI, some of the proteins do not fit to be true transporters or channels (see Supplementary Table S2).

Based on energization mechanisms, *S. indica* is endowed with a set of transporters like other fungi (Table 1) in which the most numerous groups of transport systems operate as electrochemical potential-driven transporters followed by the primary active transporters and to a lesser extent channels/pores (Table 1 and Figure 3). These distributions of mechanisms agree with a recent publication, which states that transportomes of fungi strongly evolved toward higher energetic efficiency, diminishing ATP-dependent transporters and proliferating H^+^-coupled secondary transporters (Darbani et al., 2017).

The functional classification of the transportome based on the transported substrate showed four large groups that included: (i) macromolecules, (ii) metabolic substrates and products, (iii) nutrients, and (iv) osmorregulators. It should be emphasized that this classification is only an *in silico* prediction made from the information obtained from the TCDB and by searching in the literature for the function of homologous proteins. As the activity mediated by the transporters has not been proven in this work, both the actual identity of the transported substrate and the function proposed in this work must be considered with caution. Of the four functionally different groups of transporters, the largest group was that that included macromolecule transporters that mediate the movement of proteins, lipids and RNA molecules into the cell and among the organelles (see Figure 4). Regarding nutrient transporters, as expected, a wide range of transporters appear to be responsible for ensuring the uptake and accumulation of the macronutrients C, N, P, K, S, micronutrients and vitamins that the fungus needs when it thrives in various environments.

The transcriptomic study performed here showed that during the change from a free lifestyle to symbiosis, numerous nutrient transporters were differentially regulated. In fact, this group of transporters showed the highest number of DEGs (Supplementary Table S5), among which those related to nitrogen nutrition stand out. This suggests that fungal N sources may vary in terms of the available substrate (ammonium, amino acids, or urea) or in terms of the concentrations present at different stages of plant colonization, thus activating transporters with different specificities or kinetic parameters in each case. Differential gene expression between free living conditions and those associated with the plant could denote the nutritional adaptation of the fungus in symbiosis to use compounds produced in plant metabolism. An example of a plant metabolite is urea and, as shown here, a high induction of the *DUR3* (Supplementary Table S5) homologous fungal urea transporter gene has been observed, as well as some amino acid transporters, probably readily available substrates during the growth of the fungus in the plant. The induction of the *DUR3* gene in symbiosis has also been described in previous work (Zuccaro et al., 2011). With respect to the ammonium transporters, there are two (Protein ID_73482 and Protein ID_75508), that seem to operate as efflux systems extruding the substrate out of cells. Ammonia export has previously been described as a fungal signal that triggers colonization (Fernandes et al., 2017), and interestingly, supporting this idea in this transcriptomic analysis, a clear induction of both genes after 1.5 h of infection, as well as their repression after 7 and 14 dpi has been displayed (Figure 8 and Supplementary Table S5). The expression results support the possibility that these NH_4_^+^ exporters may be involved in the transient alkalinization that has been detected in *S. indica* in the initial stages of colonization (Lanza et al., 2019), and could suggest that these transporters would be involved in signaling processes more than in N nutrition of the fungus. The fact that nutrient transporters were a numerous group regulated in symbiosis confirms previous results obtained during *S. indica* interaction with *Arabidopsis*, in which long-term biotrophic nutrition was observed after 14 dpi (Lahrmann et al., 2013).

Regarding the distribution of these transporters in the different cellular membranes, it noteworthy that the plasma membrane is the one that contains the greatest number of transporters (Figure 5) many of which are regulated in response to symbiosis (Figure 7). This suggests that the acquisition of nutrients and the extrusion of metabolic products and ions are functions that dominate the major transporter resources of fungal cells.

### About transcriptomic changes in genes encoding *Serendipita* transporters produced by moderate salinity

In this work, we have also performed a transcriptomics study of the NaCl-regulated *S. indica* transportome, as an alternative approach to the search for transporters that can mediate Na^+^ fluxes. From cultures of the fungus grown in free life in the presence of 50 mM NaCl, a relatively low response in gene expression was observed (Table 2). These results suggest that the applied saline treatment was so mild that it did not produce significant variation in expression of genes encoding transporters; or that genes encoding transporters are not easily regulated by 50 mM NaCl. The reason why this moderate salt concentration was chosen for the experiments was to clearly separate the toxic and osmotic effects of NaCl on organisms, two effects that in previous works have been demonstrated to overlap (Nivedita Rawoof et al., 2021). Despite moderate treatment, 20 transporters were regulated, and there was only one, *SiENA5,* that remained induced at all times of the experiment (Table 3), standing out as a potential salt-responsive marker gene of *S. indica.* These results agree and confirm those previously published by our group (Lanza et al., 2019). Furthermore, the gene of *SiKHA1*, known as a K^+^, H^+^ antiporter that expresses in the endoplasmic reticulum and Golgi membranes (Maresova and Sychrova, 2005), was also induced by salt treatment. It is pending to analyze its possible role of the accumulation of Na^+^ in these organelles as a mechanism of tolerance to salt. Alternatively, its induction could indicate the importance of K^+^ replenishment in these organelles to maintain adequate ionic strength for protein maturation under saline conditions (Wu et al., 2016). Among the 20 salt-regulated transporters, others involved in metabolite, sugar, or phosphate transport, were also induced by salt at some point of the experiment. Perhaps some of them could eventually carry out subsidiary fluxes of Na^+^ coupled to the transport of the substrate itself. Their real contribution to salt tolerance remains to be deciphered in future studies.

Regarding the possible role of the fungus in conferring tolerance to salt to the plants, we have observed here that during symbiosis, the transcriptional response of the fungus to NaCl was minimal and neither the *SiENA5* nor *SiKHA1* genes, which were induced by salt under free living conditions, were significantly induced in symbiosis (Table 2, see comparison NaSymb vs. Symb, 9 total DEGs, 0 DEG of transporters). At first sight, this would lead us to question whether some fungal determinant is responsible for the decrease in the Na^+^ accumulation in the plant. However, a detailed study of the 9 DEGs that responded to salt interestingly revealed that two genes involved in cell wall dynamics, one encoding a putative chitinase (Protein ID_74026) and another for a pectate-lyase (Protein ID_75472), were strongly induced (Supplementary Table S6). It has previously been described that overexpression of genes encoding fungal chitinases and plant chitinase-like proteins increases salt tolerance in plants, apparently because this induction increases cellulose content, the main component of the plant cell wall that plays a pivotal role in the regulation of salt stress (de las Mercedes Dana et al., 2006; Brotman et al., 2012; Liu et al., 2020). Furthermore, it is proposed that the amount of carboxyl groups in de-methesterified pectin could bind cations such as Na^+^ and sequester more Na^+^ in the cell wall, which could reduce Na^+^ accumulation by the plant (Byrt et al., 2018). Therefore, the induction of *S. indica* cell wall-related genes observed here could be involved, at least in part, in the Na^+^ reduction that occurs in plants during symbiosis. Its real contribution to the reduction of Na^+^ accumulated in the plant under salinity deserves further study.

The transcriptional study of the *S. indica* transportome revealed that the regulation of the transition from free living to symbiotic conditions was greater than that of living in the absence to the presence of salt, results that are in line with previous studies (Zuccaro et al., 2011; Lahrmann et al., 2013). In any case, the low transcriptional response of the fungus to moderate salt stress during the symbiosis observed here, which was even lower than that produced by exposure to salt in free living conditions, suggests that the plant protects the fungus from external conditions in such a way that the fungus hardly detects any environmental change, or if it does, it is not translated into considerable gene expression changes.

Of the 375 DEGs that were regulated by comparing fungal growth in free living conditions with symbiosis, several transporters were identified that could be involved in the maintenance of Na^+^ and K^+^ homeostasis as well as H^+^ and Ca^2+^ fluxes (Table 4). As a summary of this information, Figure 9 depicts the main ionic transporters that could have their role during symbiosis in the absence and in the presence of salt. Among them, *SiHAK1* seems to encode the most important transport system involved in K^+^ nutrition in symbiosis, regardless of the presence of salt (Table 4 and Figure 9). This transporter has previously been shown to be the major K^+^ high-affinity transport system that is strongly induced under K^+^ limiting conditions, both under free living conditions and in symbiosis (Conchillo et al., 2021). Based on our results, it is now clear that *SiHAK1* is also induced under symbiotic conditions, regardless of whether salt treatment or a K^+^ limiting stress is applied.

An interesting result observed in this work is the prominent expression of the *SiENA1* gene (Protein ID_72635) during symbiosis (Figures 9, 10 and Table 4) which possibly suggests a broader functionality of ENA-type ATPases beyond the exclusive role in salinity.

### ENA ATPases, relevant genes involved in Na and K^+^ homeostasis and in symbiosis

The results obtained here support the generally established notion that ENA ATPases seem to be the most important systems that fungi possess to deal with high NaCl concentrations (Rodríguez-Navarro and Benito, 2010). In the case of the endophyte *S. indica*, SiENA5 more than SiENA1 seems to be the main Na^+^ efflux transporter, at least when the fungus lives independently of plants. Furthermore, these results support the idea that this fungus is a typical “Na^+^-extruder” in which the main mechanism of tolerance to NaCl would be the release of Na^+^ out of cells, probably by the action of ENA ATPases as demonstrated in Lanza et al. (2019), and, apparently, as shown here, also perhaps via a Na^+^, H^+^ antiporters such as SiNHA1, rather than its accumulation in vacuoles or internal vesicles by Na^+^, H^+^ antiporters such as SiNHX1. This “Na^+^-extruder” strategy of Na tolerance is common for many other fungi that live in diverse ecosystems, including hypersaline environments (Kogej et al., 2005). However, it cannot be excluded that the secretory vesicle pathway involved in endo and/or exocytosis may participate in the reduction of Na^+^ accumulation observed in symbiosis (Figure 8 and Supplementary Table S5).

The results obtained here do not allow us to rule out that there are other fungal systems that could be involved in improving the Na^+^ tolerance of the plant, or whether the fungus induces changes in the plant that activate mechanisms that control the Na^+^ content of the plant. To shed more light on the matter, it is necessary to perform a comprehensive transcriptomics study of the plant transportome that provides complementary evidence that will allow us to resolve this pending question.

In view of our results, it can also be stated that ENA ATPases can play a relevant role during symbiosis. This claim is because the *SiENA1* gene was specifically induced during symbiosis (Figure 10), surprisingly, regardless of salt treatment, and, as demonstrated in this work, showing the highest fold change values of all DEG encoding transporters during symbiosis (Table 4). Through kinetic experiments in yeast mutants, it has been previously shown that SiENA1 also is as an K^+^ efflux transporter (Lanza et al., 2019), which raises the question of the relevance of such a function. It has been argued that the expression of a K^+^ efflux system may be interesting if the fungus faces with high K^+^ concentrations, as can occur in the biotrophic or saprotrophic stages when exposed to the cytoplasmic content of plant cells, which can reach up to 100–150 mM K^+^ (Lanza et al., 2019). In addition, *S. indica* is also known to be a particularly sensitive fungus to growth at relatively low K^+^ concentrations of around 25 mM (Conchillo et al., 2021) making it more sensitive and unable to live in the plant environment. Therefore, it is reasonable to think that the expression of a transporter that relieves cells of an excess of K^+^ could become essential for life in symbiosis. On the other hand, *SiENA1* is an alkaline pH inducible gene (Lanza et al., 2019); therefore, knowing that changes in pH can act as messengers in signal transfer (Felle, 2001), transient increases in pH that happen specifically in the plant environment may be the signals that trigger SiENA1 activity. The definitive demonstration of the relevance of SiENA1 during symbiosis must go through the acquisition of the *Siena1* mutant and the study of the effect of colonization of the plant by the mutant.

## Data availability statement

The datasets presented in this study can be found in online repositories. The names of the repository/repositories and accession number(s) can be found in the article/Supplementary material.

## Author contributions

BB and RH conceived and designed the research. BB wrote the manuscript. RH designed and carried out most of the figures and tables. ML developed the fungal growth experiments under different culture conditions and total RNA extractions for RNA-seq analysis. MA developed the computational tasks that allowed the construction of the *S. indica* transportome and part of the transcriptional analysis. ES-G and ML performed the cloning and functional characterization of SiNHA1 and SiNHX1 in yeast. All authors reviewed the manuscript and approved it for publication.

## Funding

This work was supported by grant PID2020-119441RB-I00 and AGL2016-80593-R funded by MCIN/AEI/10.13039/501100011033. Additional financial support was provided by the DGUI-UPM/CAM Research Group Program.

## Conflict of interest

The authors declare that the research was conducted in the absence of any commercial or financial relationships that could be construed as a potential conflict of interest.

## Publisher’s note

All claims expressed in this article are solely those of the authors and do not necessarily represent those of their affiliated organizations, or those of the publisher, the editors and the reviewers. Any product that may be evaluated in this article, or claim that may be made by its manufacturer, is not guaranteed or endorsed by the publisher.

## Footnotes

^1^ http://www.tcdb.org/search/index.php?query=&type=system

^2^ https://mycocosm.jgi.doe.gov/mycocosm/annotations/browser/tcdb/summary;uL94hn?p=Pirin1

^3^ http://eggnog5.embl.de/#/app/home

^4^ https://genome.jgi.doe.gov/Pirin1/Pirin1.home.html

^5^ https://mycocosm.jgi.doe.gov/Pirin1/Pirin1.home.html

^6^ https://mycocosm.jgi.doe.gov/mycocosm/annotations/browser/tcdb/summary;AC_HVU?p=Pirin1

^7^ http://www.membranetransport.org/transportDB2/index.html

^8^ https://www.tcdb.org/
