# Supplementary figures and images for: The transportome of the endophyte Serendipita indica in free life and symbiosis with Arabidopsis and its expression in moderate salinity
Source: Front Microbiol. 2023 Jun 19;14:1191255. doi: 10.3389/fmicb.2023.1191255 (PMC10315484; doi:10.3389/fmicb.2023.1191255)

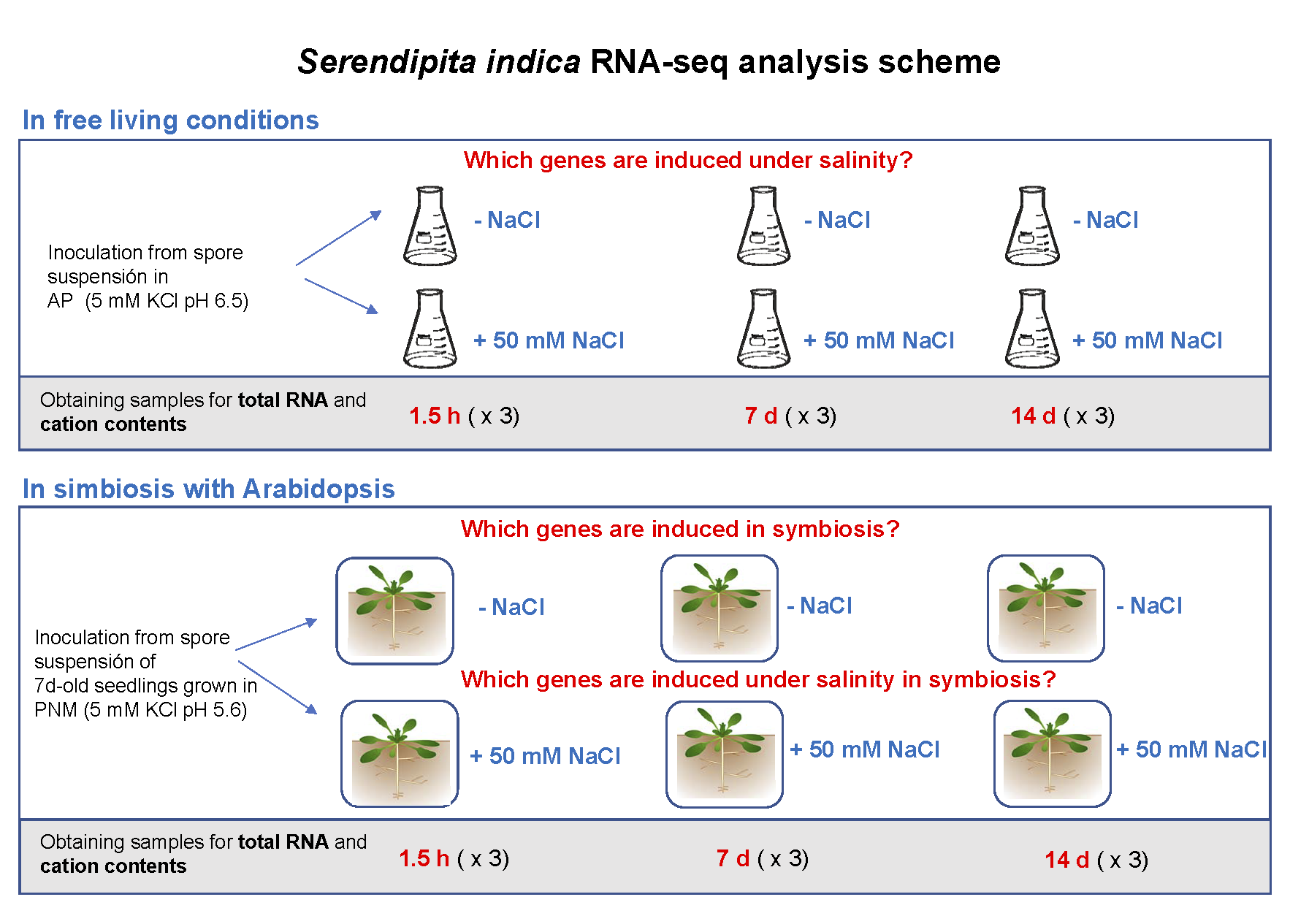

Supplement: Supplementary file 1 [file Image_1.TIF]

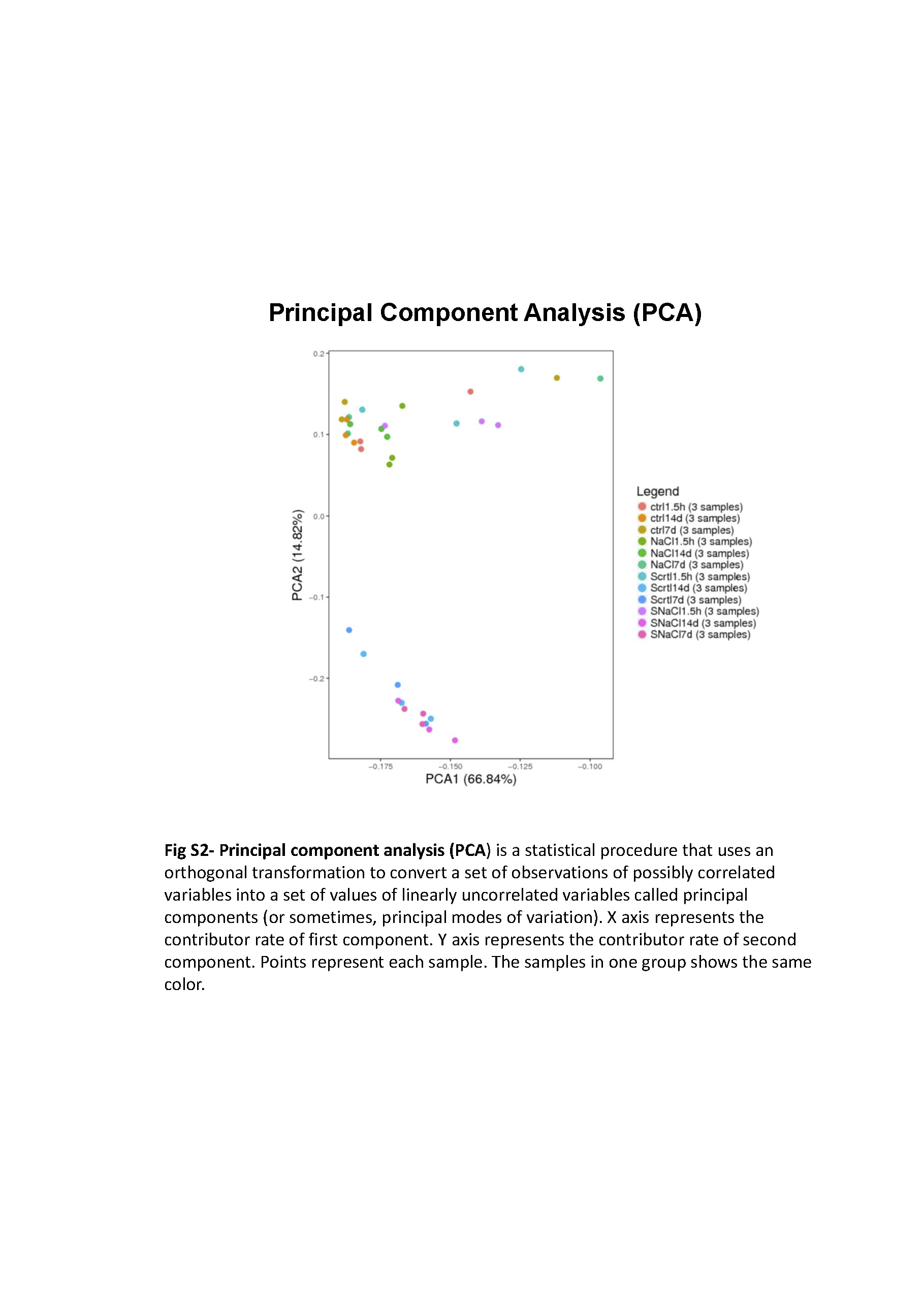

Supplement: Supplementary file 2 [file Image_2.TIFF]

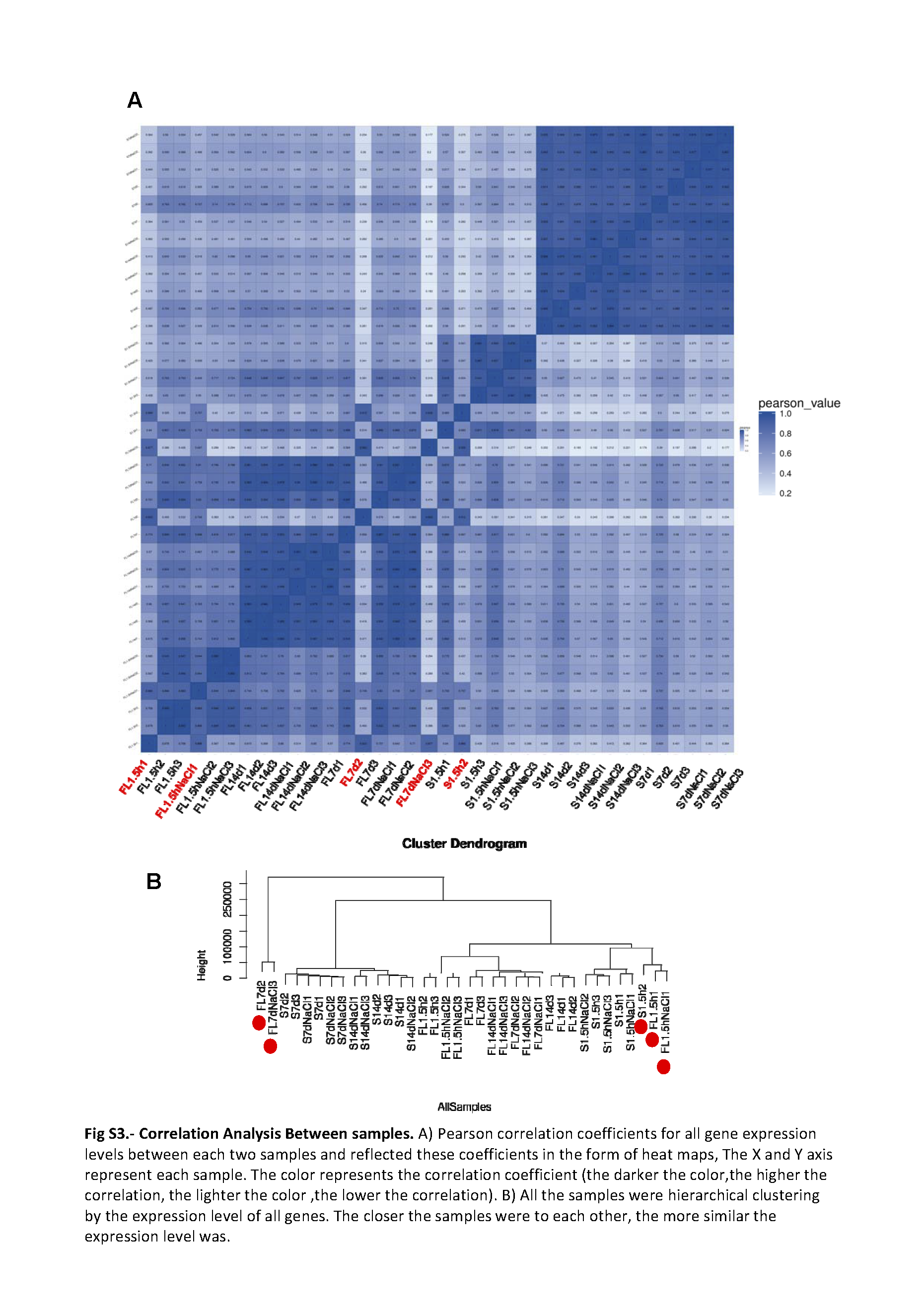

Supplement: Supplementary file 3 [file Image_3.TIF]

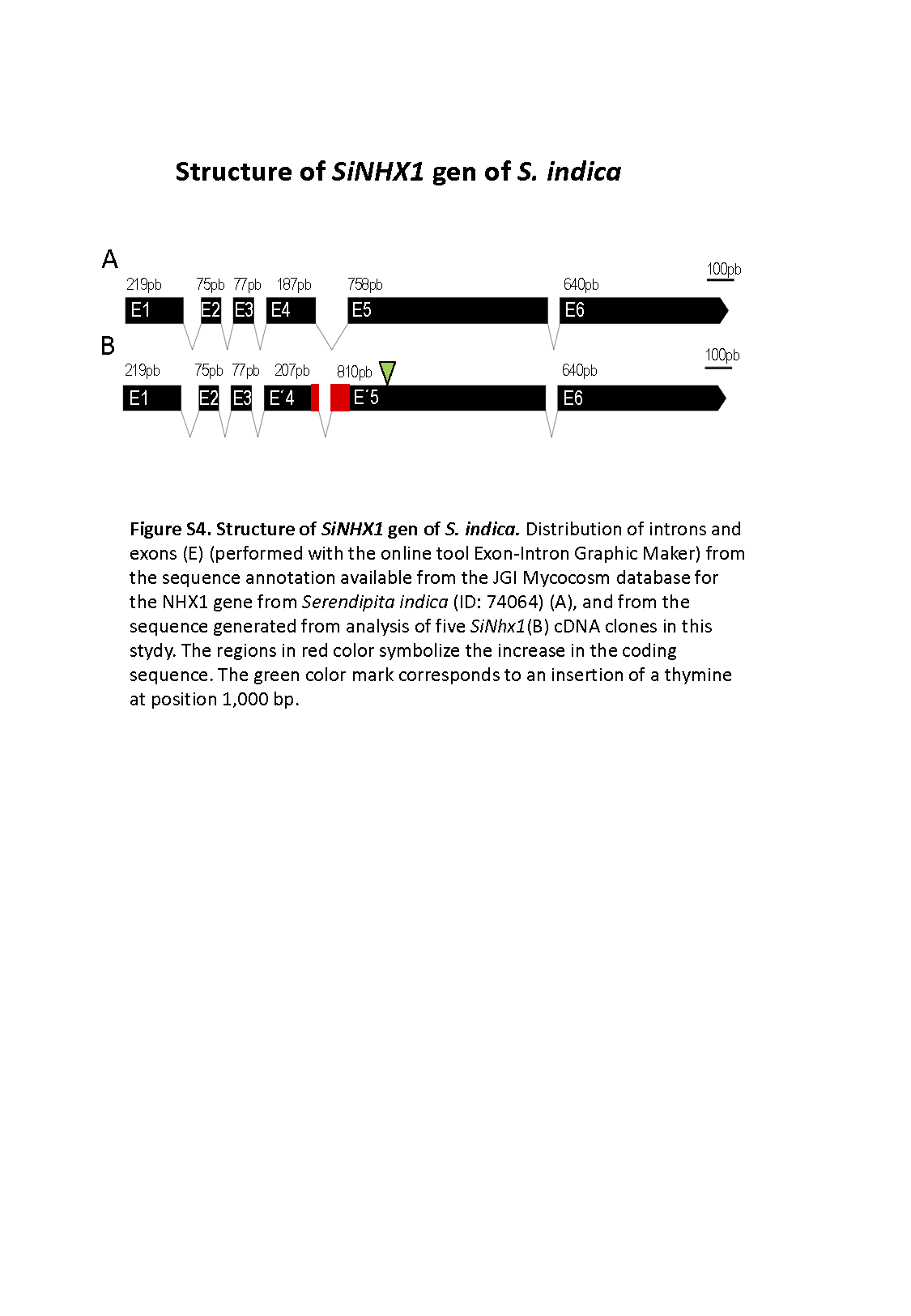

Supplement: Supplementary file 4 [file Image_4.TIF]
